# Entropy generation and induced magnetic field in pseudoplastic nanofluid flow near a stagnant point

**DOI:** 10.1038/s41598-021-02997-3

**Published:** 2021-12-09

**Authors:** Enran Hou, Azad Hussain, Aysha Rehman, Dumitru Baleanu, Sohail Nadeem, R. T. Matoog, Ilyas Khan, El-Sayed M. Sherif

**Affiliations:** 1 College of Mathematics, Huaibei Normal University, Huaibei, 235000 China; 2 Department of Mathematics, University of Gujrat, Gujrat, 50700 Pakistan; 3 Department of Mathematics, Cankaya University, Ankara, Turkey; 4 Institute of Space Sciences, 077125 Magurele, Romania; 5 Department of Mathematics, Quaid-I-Azam University, Islamabad, 44000 Pakistan; 6 Department of Mathematics, Faculty of Applied Sciences, Umm Al-Qura University, Makkah, Saudi Arabia; 7 Department of Mathematics, College of Science Al-Zulfi, Majmaah University, Al-Majmaah, 11952 Saudi Arabia; 8 Department of Mechanical Engineering, College of Engineering, King Saud University, P.O. Box 800, Al-Riyadh, 11421 Saudi Arabia

**Keywords:** Mathematics and computing, Nanoscience and technology

## Abstract

In this present article the entropy generation, induced magnetic field, and mixed convection stagnant point flow of pseudoplastic nano liquid over an elastic surface is investigated. The Buongiorno model is employed in modeling. Through the use of the boundary layer idea, flow equations are transformed from compact to component form. The system of equations is solved numerically. The Induced magnetic spectrum falls near the boundary and grows further away as the reciprocal of the magnetic Prandtl number improves. The fluctuation of induced magnetic rises while expanding the values of mixed convection, thermophoresis, and magnetic parameters, whereas it declines for increment in the Brownian and stretching parameters. The velocity amplitude ascends and temperature descends for the rise in magnetic parameter. The mass transfer patterns degrade for the higher amount of buoyancy ratio while it boosts by the magnification of mixed convection and stretching parameters. Streamlines behavior is also taken into account against the different amounts of mixed convection and magnetic parameters. The pseudoplastic nanofluids are applicable in all electronic devices for increasing the heating or cooling rate in them. Further, pseudoplastic nanofluids are also applicable in reducing skin friction coefficient.

## Introduction

Many scientists are investigating the impacts of MHD on electrically conductive and viscous, fluid because such type of issues competes with numerous industrial techniques such as liquid metal fast reactor (LMFR), flight propulsion systems, energy generation, thermonuclear fusion, crude oil purification, and plasma confinement^1^. In partly ionized liquids and metallic liquids, the induced magnetic field created by fluid motion is minimal due to its little magnetic Reynolds number. Still, the induced magnetic field performs a very important part when the magnetic amount of Reynolds is higher than or equal to one and should be taken into consideration. Some of the procedures in which the impact of the induced magnetic field is important are the liquid flow in star formation, rotating magnetic stars, solar dynamo, planetary issues, earth's interior, and fusion applications with plasma containment. Because of the different uses of the induced magnetic field, we took such liquids with a sufficiently big magnetic amount of Reynolds^1^. Boundary layer flow by stretching surfaces has been the subject of broad study because of their vast scope of uses, for example, assembling of nourishment and paper polymer expulsion, glass fiber generation, wire drawing, extending of plastic films, and numerous others^2^. The application of the induced magnetic field may be seen in Refs.^3–9^.

Natural convection is heat variation at distinct liquid locations, but forced convection is defined as warmth caused by certain outside forces. Mixed convection, however, is a mixture of forced and free convection. Numerous applications of blended convection in the real world are fan-cooled electronic equipment, heat exchangers^10^. Ali et al.^11^ discussed viscous, steady stagnation point magnetohydrodynamic (MHD), combined convection flow of incompressible, and electric fluid on a vertical flat plate with the impact of the induced magnetic field. Kumari et al.^12^ demonstrated the steady, blended convection and MHD flow of a viscoelastic liquid near a two-dimensional stagnant point with a magnetic field on the Maxwell (UCM) upper-convected fluid model. Ali et al.^13^ performed a stability assessment on a dynamic magnetohydrodynamic (MHD) blended convection fluid flow on a surface and the impact of the induced magnetic field is also taken into account. Ahmad et al.^14^ explored numerical studies of the chemical reaction of ionized liquid flow towards a plate with the induced magnetic field. Raju et al.^15^ described the impact of varying temperature conductivity and induced magnetic field over an unstable two-dimensional channel flow of Jeffrey's incompressible laminar blended convective and chemically reacted fluid embedded with a non-Darcy porous medium. Turkyilmazoglu et al.^16^ focused on the combined study of the MHD viscous flow due to a nonlinear deforming body having a uniform magnetic field with either heat absorption or generation. Rajendrappa et al.^17^ scrutinized the impact of viscosity on the squeeze film characteristics between porous circular sheets lubricated with non-Newtonian fluids. Lee et al.^18^ explored the study flow of Power-Law Fluids in a circular tube. Sadeghi et al.^19^ investigated the heat behavior and buoyancy-driven magnetic flow in ferrofluid with two cylinders. Takhar et al.^20^ discussed the impact of the magnetic field in mixed convection unsteady flow from a rotating vertical cone.

Today, due to the manufacturing of environmental prolusions and the consumption of irreversible energy sources, the optimization of heat transfer processes through the use of cooling liquids has become very essential in various sectors, such as aerospace, energy generation, transportation, petrochemicals, electronics, and machining. To achieve high efficiency, the heat transfer device requires reduced dimension and enhanced heat transfer in each surface area unit. In recent decades, the advancement of technology, enhancement in the rheological characteristics of cooling liquids, and the generation of solid–fluid suspensions called nanofluid to make heat exchangers and industrial tools and improve their thermal efficiency^21^. Many scientists have indicated, that heat transfer increased through nanofluids^22–40^.

One of the most significant subclasses of rheological fluid models is the Carreau-Yasuda model, an extended form of Carreau^41^ improved by Yasuda^42^. Khan et al.^43^ the study of the effect of improving Carreau-Yasuda fluid diffusion on a rotating disk with slip circumstances was explored. Khan et al.^44^ discovered Darcy-Forchheimer impact on Carreau -Yasuda magnetohydrodynamics nanofluid flow. Seyyedi et al.^45^ explored a square inclined cavity with entropy optimization.

The present investigation's goal is to investigate the influence of entropy production, and the combined convection flow of pseudoplastic nano liquid over a stretchable sheet with the impact of an induced magnetic field by applying a model proposed by Buongiorno^46^. In our point of view, the problem is new and original. Therefore, the present research is the first attempt to use the induced magnetic field with combined convection pseudoplastic non-Newtonian nanofluid flow to investigate mass and heat transport behavior on the vertical stretched plate in the existence of stagnation point using MATLAB bvp4c algorithm. The outcomes of effective parameters such as Brownian motion, magnetic, buoyancy ratio, mixed convection, stretching, thermophoresis, reciprocal of the magnetic Prandtl, Weissenberg, Lewis, and Prandtl numbers on the mass and heat transport characteristics are examined and illustrated graphically. The analysis made in this article shows that the mass and thermal transport rates are improved in the flow of pseudoplastic non-Newtonian nanofluid.

## Mathematical equations

**Figure 1:**
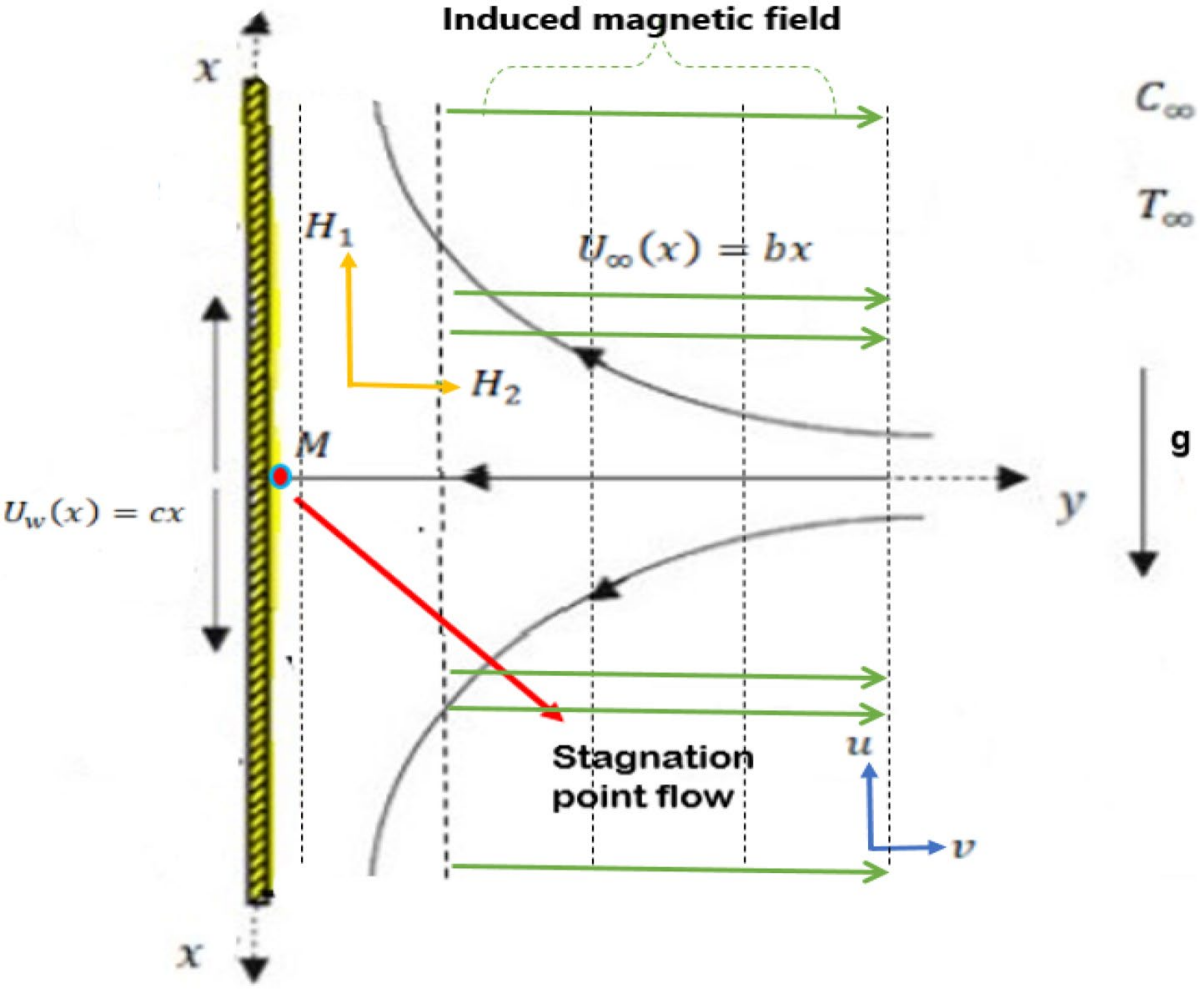
Schematic flow configuration.

Considers the steady, two-dimensional, incompressible flow with the impact of the induced magnetic field and combined convection pseudoplastic stagnation point nanofluid towards a stretched sheet as shown in Fig. 1. Two similar and contrary forces are applied to the surface along the x-axis in a manner the sheet stretched with velocity ${U}_{w}(x)=cx$ and ambient liquid velocity is ${U}_{\infty }(x)=bx$ while the origin is fixed at M, see Fig. 1. The sheet is heated by convection from a hot fluid at a temperature ${T}_{w}$ which is by heat transfer coefficient ${h}_{f}$. Consider fluid flow velocity will change through $x$ and $y$ axis in a manner that the $y-axis$ is taken horizontally and the $x-axis$ is taken vertically. The fundamental equation of pseudoplastic fluid is^47^

1 $$\tau =\left[{\mu }_{\infty }+\left({\mu }_{0}-{\mu }_{\infty }\right){({(1+\Gamma \stackrel{\cdot }{\gamma })}^{d})}^{\frac{n-1}{d}}\right]\text{\hspace{0.17em}}{A}_{1},$$

here ${\mu }_{\infty }$ represents infinite shear rate viscosity, ${\mu }_{0}$ denotes zero shear rate viscosity, $n$, $d$, and $\Gamma$ are fluid characteristics, $\tau$ is extra stress tensor, ${A}_{1}$ is labelled as first Rivlin Ericksen tensor and $\stackrel{\cdot }{\gamma }$ is expressed via $\stackrel{\cdot }{\gamma }=\sqrt{tr({A}_{1}^{2})\frac{1}{2}}$ , here ${A}_{1}=\left[{(gradv)}^{t}+gradv\right]$. Considering that infinite shear rate viscosity ${\mu }_{\infty }=0$ and then Eq. (1) in the following form

2 $$\tau =\left[{\mu }_{0}{({(1+\Gamma \stackrel{\cdot }{\gamma })}^{d})}^{\frac{n-1}{d}}\right]\text{\hspace{0.17em}}{A}_{1}.$$

The governing equations are as follows

3 $$\frac{\partial u}{\partial x}+\frac{\partial v}{\partial y}=0,$$

4 $$\frac{\partial {H}_{1}}{\partial x}+\frac{\partial {H}_{2}}{\partial y}=0,$$

5 $$\begin{array}{c}u\frac{\partial u}{\partial x}+v\frac{\partial u}{\partial y}-\frac{{\mu }_{\infty }}{4\pi {\rho }_{f}}({H}_{1}\frac{\partial {H}_{1}}{\partial x}+{H}_{2}\frac{\partial {H}_{1}}{\partial y})=({U}_{\infty }\frac{d{U}_{\infty }}{dx}-\frac{{\mu }_{\infty }{H}_{\infty }}{4\pi {\rho }_{f}}\frac{d{H}_{\infty }}{dx})+\nu \frac{{\partial }^{2}u}{\partial {y}^{2}}\\ +\nu \left[\frac{(n-1)}{d}(d+1){\Gamma }^{d}{\left(\frac{\partial u}{\partial y}\right)}^{d}\frac{{\partial }^{2}u}{\partial {y}^{2}}\right]\text{\hspace{0.17em}}\\ +g\left[\frac{(1-C)\beta {\rho }_{fm}}{{\rho }_{f}}(T-{T}_{\infty })-\left(\frac{{\rho }_{p}-{\rho }_{fm}}{{\rho }_{f}}\right)(C-{C}_{\infty })\right],\end{array}$$

6 $$u\frac{\partial {H}_{1}}{\partial x}+v\frac{\partial {H}_{1}}{\partial y}-{H}_{1}\frac{\partial u}{\partial x}-{H}_{2}\frac{\partial u}{\partial y}={\alpha }_{1}^{*}\frac{{\partial }^{2}{H}_{1}}{\partial {y}^{2}},$$

7 $$u\frac{\partial T}{\partial x}+v\frac{\partial T}{\partial y}=\frac{k}{{\left(\rho {c}_{p}\right)}_{f}}\frac{{\partial }^{2}T}{\partial {y}^{2}}+\frac{{(\rho {c}_{p})}_{p}}{{(\rho {c}_{p})}_{f}}\left[{D}_{B}\frac{\partial C}{\partial y}\frac{\partial T}{\partial y}+\left(\frac{{D}_{T}}{{T}_{\infty }}\right)\text{\hspace{0.17em}}{\left(\frac{\partial T}{\partial y}\right)}^{2}\right],$$

8 $$u\frac{\partial C}{\partial x}+v\frac{\partial C}{\partial y}={D}_{B}\frac{{\partial }^{2}C}{\partial {y}^{2}}+\left(\frac{{D}_{T}}{{T}_{\infty }}\right)\text{\hspace{0.17em}}\frac{{\partial }^{2}T}{\partial {y}^{2}}.$$

where $\left({H}_{1},\text{\hspace{0.17em}}{H}_{2}\right)$ and $\left(u,\text{\hspace{0.17em}}v\right)$ describe the magnetic field and velocity components along the $x$ and $y$ directions, respectively, whereas ${U}_{w}(x)=cx$ and ${H}_{\infty }\left(x\right)=x{H}_{0}$ are the $x$ velocity and $y$ magnetic field at the edge of the boundary layer and ${H}_{0}$ is the uniform value of the vertical magnetic field at the infinity upstream.

The invoking boundaries are,

9 $$u={U}_{w}(x)=cx,\text{\hspace{0.17em}} \, v=0,\text{\hspace{0.17em} }\frac{\partial {H}_{1}}{\partial y}=0,\text{\hspace{0.17em} }{H}_{2}=0,\text{\hspace{0.17em}}T\to {T}_{w}\text{, }C\to {C}_{w}\text{ at }y\to 0,$$

10 $$u={U}_{\infty }(x)=bx,\text{\hspace{0.17em} }{H}_{1}={H}_{\infty },\text{\hspace{0.17em}} \, T\to {T}_{\infty },\text{\hspace{0.17em}}C\to {C}_{\infty },\text{\hspace{0.17em}}\text{ at }y\to \infty .$$

Suitable similarity transformations are defined as,

11 $$\psi =\sqrt{c\nu }xF\left(\eta \right),\text{\hspace{0.17em}} \, \eta =\sqrt{\frac{c}{\nu }}y,\text{\hspace{0.17em}} \, u=\frac{\partial \psi }{\partial y}=cx{F}^{^{\prime}}\left(\eta \right),\text{\hspace{0.17em}} \, v=-\frac{\partial \psi }{\partial x}=-\sqrt{c\nu }F\left(\eta \right), {H}_{1}=\left(\frac{{H}_{0}x}{L}\right)\text{\hspace{0.17em}}{g}_{1}^{{^{\prime}}}\left(\eta \right), {H}_{2}=-\sqrt{\left(\frac{\nu }{c}\right)}\left(\frac{{H}_{0}}{L}\right)\text{\hspace{0.17em}}{g}_{1}\left(\eta \right),{H}_{\infty }={H}_{0}\left(x/L\right), \theta (\eta )=\frac{T-{T}_{\infty }}{{T}_{w}-{T}_{\infty }},\text{\hspace{0.17em}} \, \varphi (\eta )=\frac{C-{C}_{\infty }}{{C}_{w}-{C}_{\infty }}.$$

The magnetized pressure is described as

12 $$p=p+\frac{\mu {\left|H\right|}^{2}}{8\pi }$$

Consequently, Eqs. (3) and (4) are satisfied identically. Equations (5–8) and Eqs. (9) and (10) reduce to

13 $$ \left[ {1 + \frac{{\left( {n - 1)} \right)}}{d}\left( {d + 1} \right)W_{e}^{d} \left( {F^{\prime\prime}} \right)^{d} } \right]F^{\prime\prime\prime} + F^{\prime\prime} - \left( {F^{\prime}} \right)^{2}  + \varepsilon ^{2}  + \beta \left[ {\left( {g^{\prime}_{1} } \right)^{2}  - g_{1} g^{\prime\prime}_{1} } \right] - 1 + \lambda \theta  - N_{r} \varphi  = 0,  $$

14 $${\alpha }_{1}{g}_{1}^{{^{\prime}}{^{\prime}}{^{\prime}}}+F{g}_{1}^{{^{\prime}}}-{g}_{1}{F}^{{^{\prime}}{^{\prime}}}=0,$$

15 $$ \frac{1}{{\Pr }}\theta ^{\prime\prime} + \theta ^{\prime}F + Nb\varphi ^{\prime}\theta ^{\prime} + Nt\left( {\theta ^{\prime}} \right)^{2}  = 0, $$

16 $$ \varphi ^{\prime\prime} + Le\Pr F\varphi ^{\prime} + \frac{{N_{t} }}{{N_{b} }}\theta ^{\prime\prime} =0,$$

with boundaries

17 $$F=0\text{, }{F}^{^{\prime}}=1\text{, }\theta =1\text{, }\varphi =1\text{, }{g}_{1}=0\text{, }{g}_{1}^{^{\prime\prime} }=0\text{ at }\eta \to 0,$$

18 $${F}^{^{\prime}}=\varepsilon \text{, }\theta =0\text{, }\varphi =0\text{, }{g}_{1}^{{^{\prime}}}=1\text{ at }\eta \to \infty .$$

Here, prime denotes derivative for $\eta ,$ and other dimensionless characteristics are defined as

19 $$\begin{array}{ccc}\lambda =\frac{(1-C)\beta {\rho }_{fm}({T}_{w}-{T}_{\infty })g}{c{U}_{w}{\rho }_{f}},& {N}_{r}=\frac{\left({\rho }_{p}-{\rho }_{fm}\right)({C}_{w}-{C}_{\infty })g}{c{U}_{w}{\rho }_{f}},& \varepsilon =\frac{b}{c},\\ {W}_{e}^{d}={\left(\frac{{c}^{1/2}{U}_{w}\Gamma }{\sqrt{\nu }}\right)}^{d},& {N}_{t}=\frac{{\left(\rho {c}_{p}\right)}_{p}{D}_{T}({T}_{w}-{T}_{\infty })}{{\left(\rho {c}_{p}\right)}_{f}\nu {T}_{\infty }},& \text{Pr}=\frac{\nu }{\alpha },\\ {N}_{b}=\frac{{\left(\rho {c}_{p}\right)}_{p}{D}_{B}({C}_{w}-{C}_{\infty })}{{\left(\rho {c}_{p}\right)}_{f}\nu },& Le=\frac{\alpha }{{D}_{B}},& \alpha =\frac{K}{{\left(\rho {c}_{p}\right)}_{f}},\\ {\alpha }_{1}=\frac{{\alpha }_{1}^{*}}{\nu },& \beta =\frac{{H}_{0}^{2}{\mu }_{\infty }}{4{c}^{2}\pi {\rho }_{f}}.& \end{array}$$

The local Sherwood number $S{h}_{x}$, local Nusselt number $N{u}_{x}$ , skin friction coefficient ${C}_{f}$ are,

20 $${C}_{f}=\frac{{\tau }_{w}}{\rho {U}_{w}^{2}},\text{\hspace{0.17em}} \, N{u}_{x}=\frac{x{q}_{w}}{k({T}_{w}-{T}_{\infty })},\text{\hspace{0.17em}} \, S{h}_{x}=\frac{x{q}_{m}}{{D}_{B}({C}_{w}-{C}_{\infty })},$$

where ${q}_{w}$ presents surface heat flux, ${\tau }_{w}$ denotes surface shear stress, and ${q}_{m}$ denotes surface mass flux for Carreau-Yasuda fluid is

21 $${\tau }_{w}={\left[\mu \left(1+\left(\frac{n-1}{d}\right)\text{\hspace{0.17em}}{\Gamma }^{d}{\left(\frac{\partial u}{\partial y}\right)}^{d}\right)\text{\hspace{0.17em}}\frac{\partial u}{\partial y}\right]}_{y=0},$$

22 $${q}_{w}=-k{\left[\frac{\partial T}{\partial y}\right]}_{y=0},$$

and

23 $${q}_{m}=-{D}_{B}{\left[\frac{\partial C}{\partial y}\right]}_{y=0}.$$

After using similarity transformations Eq. (11) the expression for dimensionless local Sherwood number, skin friction, and local Nusselt number becomes

24 $$ C_{f} \text{Re} _{x}^{{1/2}}  = \left[ {f^{\prime\prime}(0) + \left( {\frac{{(n - 1)}}{d}} \right)W_{e}^{d} f^{\prime\prime}(0)^{{d + 1}} } \right], $$

25 $$N{u}_{x}R{e}_{x}^{-1/2}=-{\theta }^{^{\prime}}(0),$$

and

26 $$S{h}_{x}R{e}_{x}^{-1/2}=-{\varphi }^{^{\prime}}\left(0\right),$$

where

$$R{e}_{x}^{1/2}=\sqrt{\frac{{U}_{w}x}{\nu }}.$$

## Modeling of entropy

The entropy in dimensional form for the pseudoplastic fluid is defined as

27 $$SG=\frac{k}{{T}_{\infty }^{2}}{(\frac{\partial T}{\partial y})}^{2}+\frac{{\mu }_{0}}{{T}_{\infty }}\Psi +\frac{RD}{{C}_{\infty }}{(\frac{\partial C}{\partial y})}^{2}+\frac{RD}{{T}_{\infty }}(\frac{\partial C}{\partial y}\frac{\partial T}{\partial y}),$$

28 $$\Psi ={(\frac{\partial u}{\partial y})}^{2}+{(\frac{\partial u}{\partial y})}^{2}{(\frac{\partial u}{\partial y})}^{d}(\frac{n-1}{d}){\Gamma }^{d}.$$

Invoking Eq. (28) in Eq. (27),

29 $$SG=\frac{k}{{T}_{\infty }^{2}}{(\frac{\partial T}{\partial y})}^{2}+\frac{{\mu }_{0}}{{T}_{\infty }}\left[{(\frac{\partial u}{\partial y})}^{2}+{(\frac{\partial u}{\partial y})}^{2}{(\frac{\partial u}{\partial y})}^{d}(\frac{n-1}{d}){\Gamma }^{d}\right]+\frac{RD}{{C}_{\infty }}{(\frac{\partial C}{\partial y})}^{2}+\frac{RD}{{T}_{\infty }}(\frac{\partial C}{\partial y}\frac{\partial T}{\partial y}).$$

By using similarity transformation Eq. (11) in Eq. (29).

30 $$NG={\delta }_{1}{\left({\theta }^{^{\prime}}\right)}^{2}+Br{\left({F}^{{^{\prime}}{^{\prime}}}\right)}^{2}\left[1+\left(\frac{n-1}{d}\right)\text{\hspace{0.17em}}{\left({W}_{e}\right)}^{d}{\left({F}^{^{\prime\prime} }\right)}^{d}\right]+\frac{{\delta }_{2}}{{\delta }_{1}}L{\left({\varphi }^{{^{\prime}}}\right)}^{2}+L{\theta }^{{^{\prime}}}{\varphi }^{{^{\prime}}},$$

where ${\delta }_{2}$ presents concentration difference variable, ${\delta }_{1}$ difference variable, $L$ the diffusion characteristic, $NG$ local rate of entropy generation and $Br$ the Brinkman number,

31 $${\delta }_{1}=\frac{\Delta T}{{T}_{\infty }}=\frac{({T}_{w}-{T}_{\infty })}{{T}_{\infty }},\text{\hspace{0.17em}} {\delta }_{2}=\frac{\Delta C}{{C}_{\infty }}=\frac{({C}_{w}-{C}_{\infty })}{{C}_{\infty }}, L=\frac{RD\Delta C}{k}, Br=\frac{{\mu }_{0}{c}^{2}{x}^{2}}{k\Delta T},NG=\frac{SG\nu {T}_{\infty }}{k\Delta TC}$$

The Bejan number is

32 $$Be=\frac{\text{Heat transfer irreversibility+Mass transfer irreversibility}}{\text{Total entropy}}$$

33 $$Be=\frac{{\delta }_{1}{\left({\theta }^{^{\prime}}\right)}^{2}+\frac{{\delta }_{2}}{{\delta }_{1}}L{\left({\varphi }^{{^{\prime}}}\right)}^{2}+L{\theta }^{{^{\prime}}}{\varphi }^{{^{\prime}}}}{{\delta }_{1}{\left({\theta }^{{^{\prime}}}\right)}^{2}+Br{\left({F}^{{^{\prime}}{^{\prime}}}\right)}^{2}\left[1+\left(\frac{n-1}{d}\right)\text{\hspace{0.17em}}{\left({W}_{e}\right)}^{d}{\left({F}^{{^{\prime}}{^{\prime}}}\right)}^{d}\right]+\frac{{\delta }_{2}}{{\delta }_{1}}L{\left({\varphi }^{{^{\prime}}}\right)}^{2}+L{\theta }^{{^{\prime}}}{\varphi }^{{^{\prime}}}}$$

## Results and discussion

**Figure 2:**
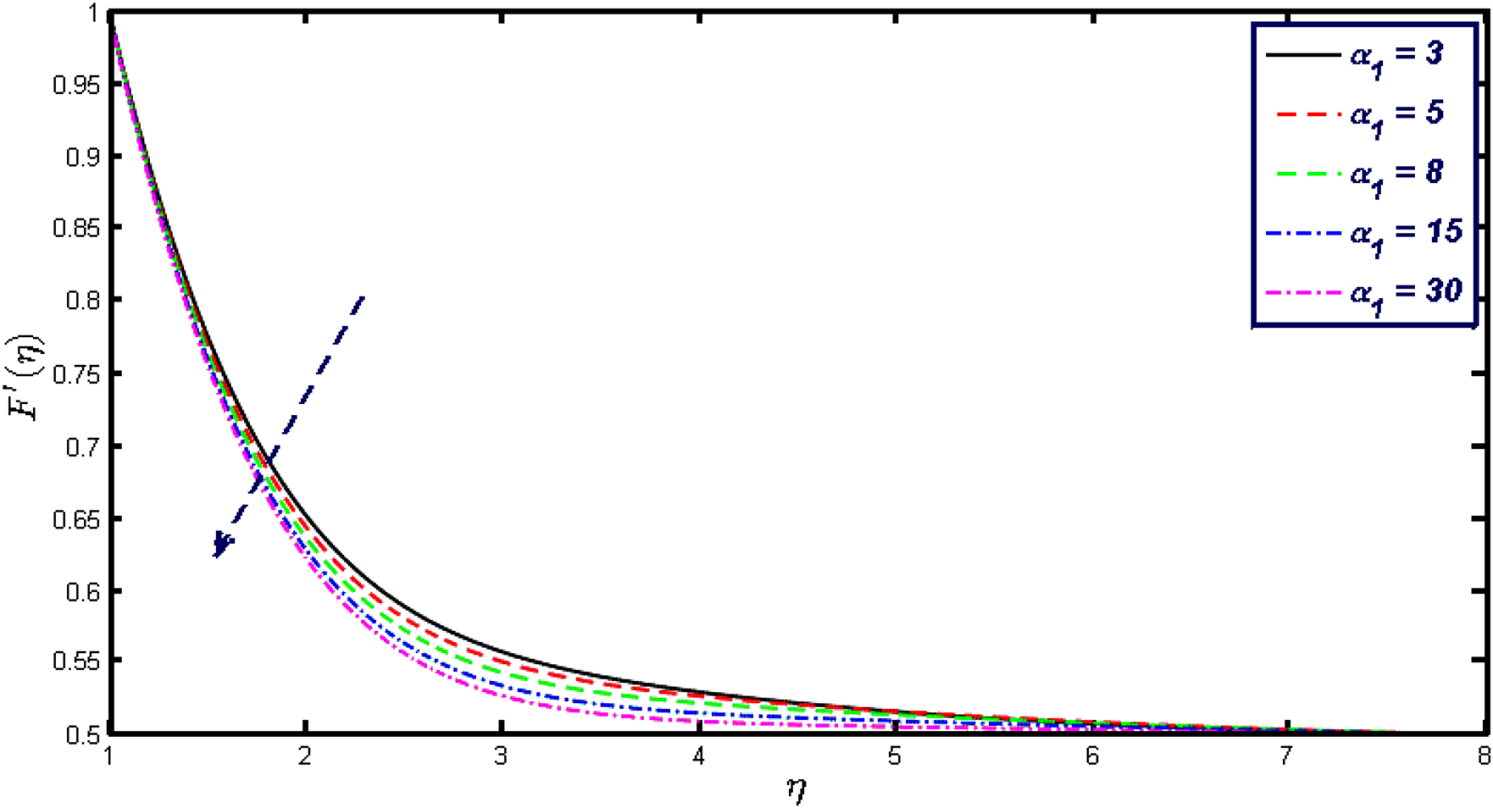
Velocity profile for different values of ${\alpha }_{1}.$

**Figure 3:**
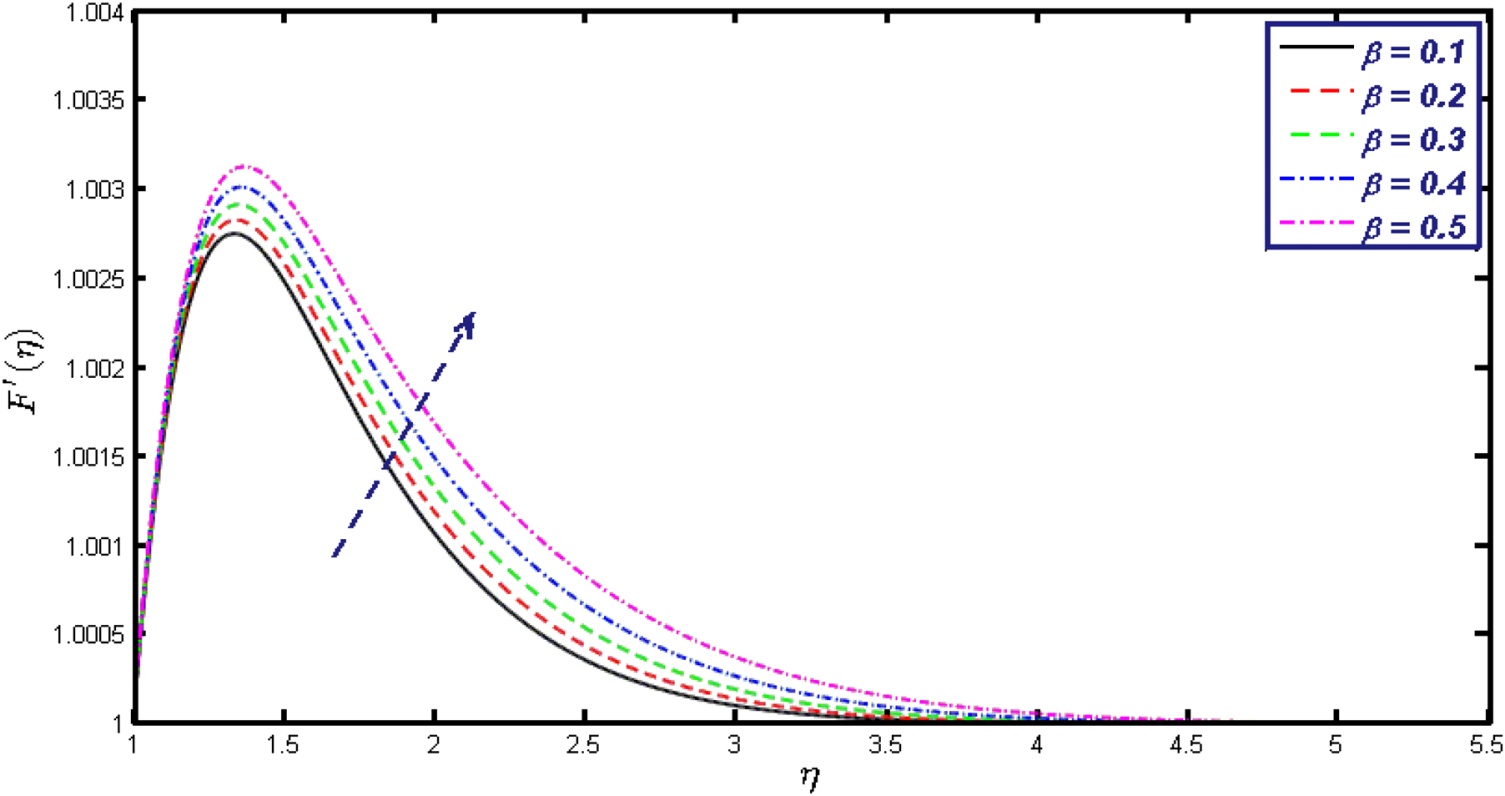
Velocity field for different values of $\beta .$

**Figure 4:**
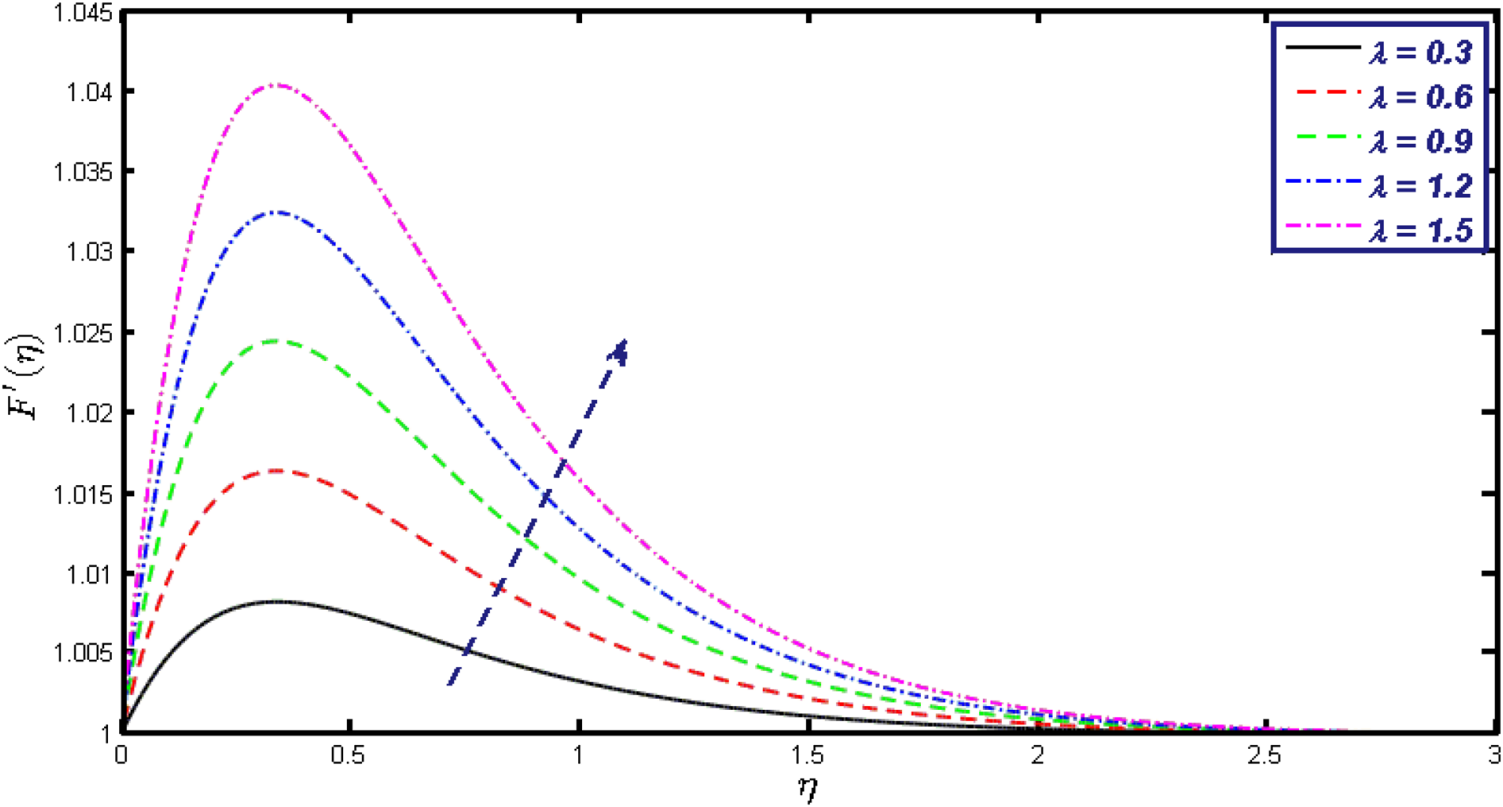
Velocity field for different values of $\lambda .$

**Figure 5:**
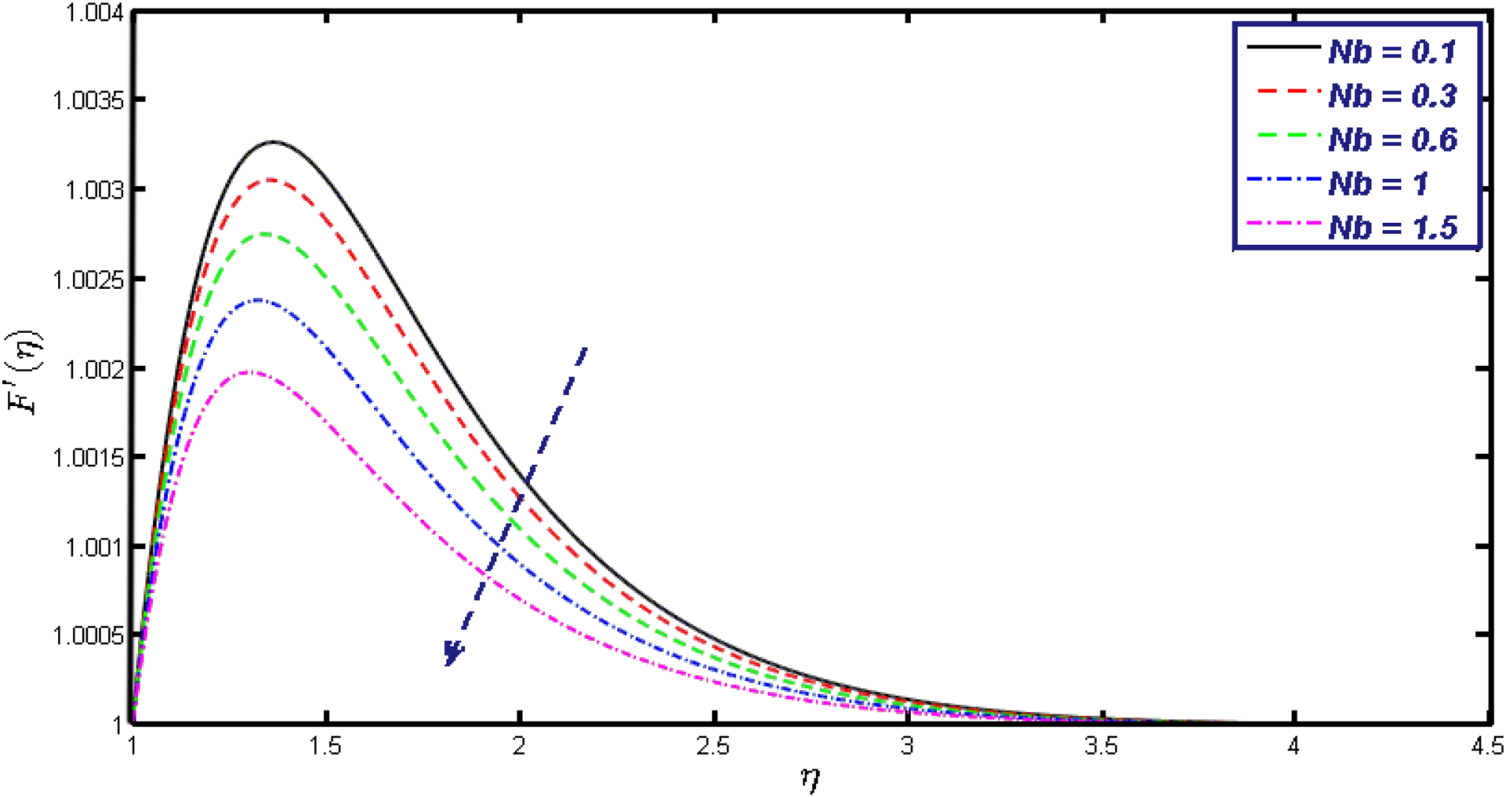
Velocity field for different values of $Nb.$

**Figure 6:**
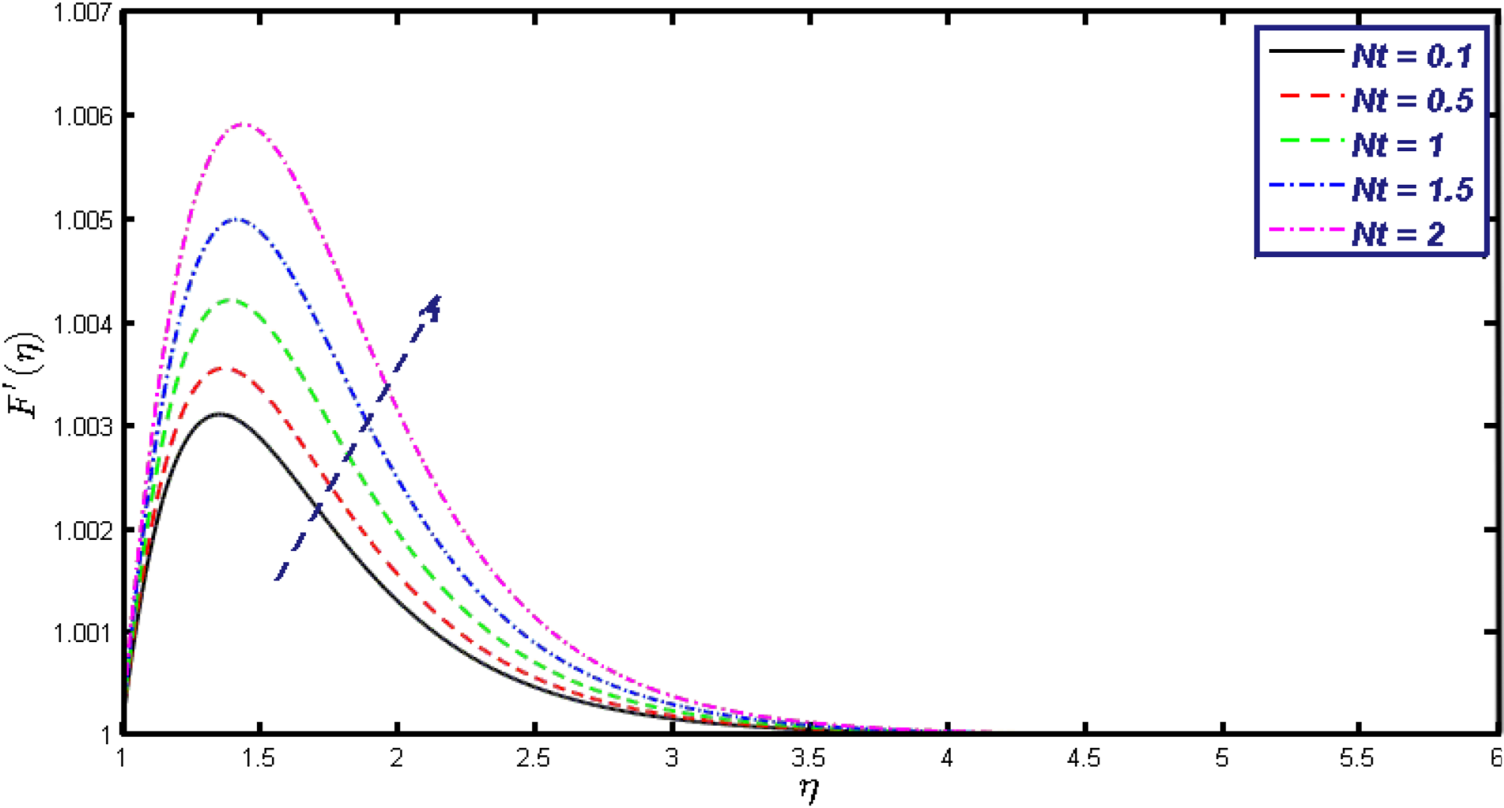
Velocity field for different values of $Nt.$

**Figure 7:**
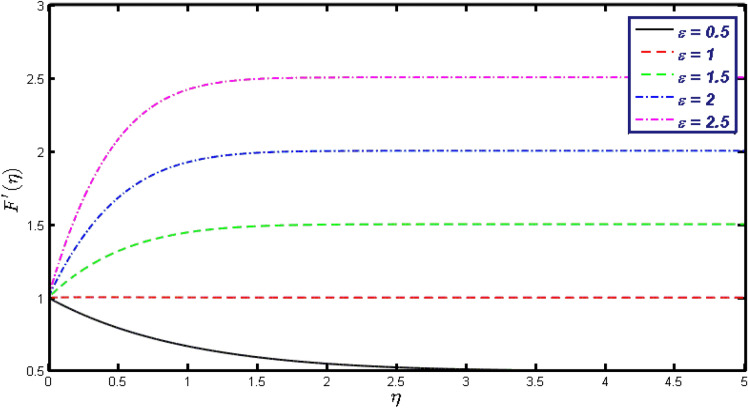
Velocity field for different values of $\varepsilon$.

**Figure 8:**
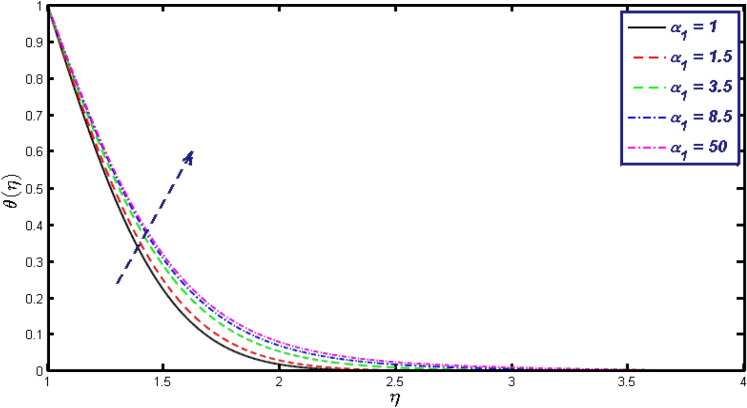
Temperature field for different values of ${\alpha }_{1}.$

**Figure 9:**
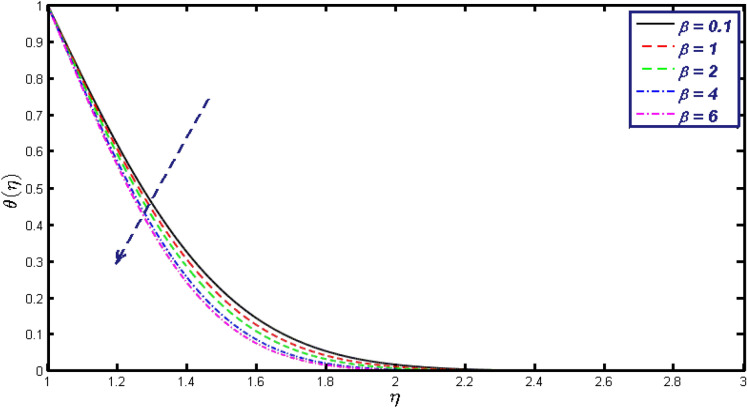
Temperature field for different values of $\beta .$

**Figure 10:**
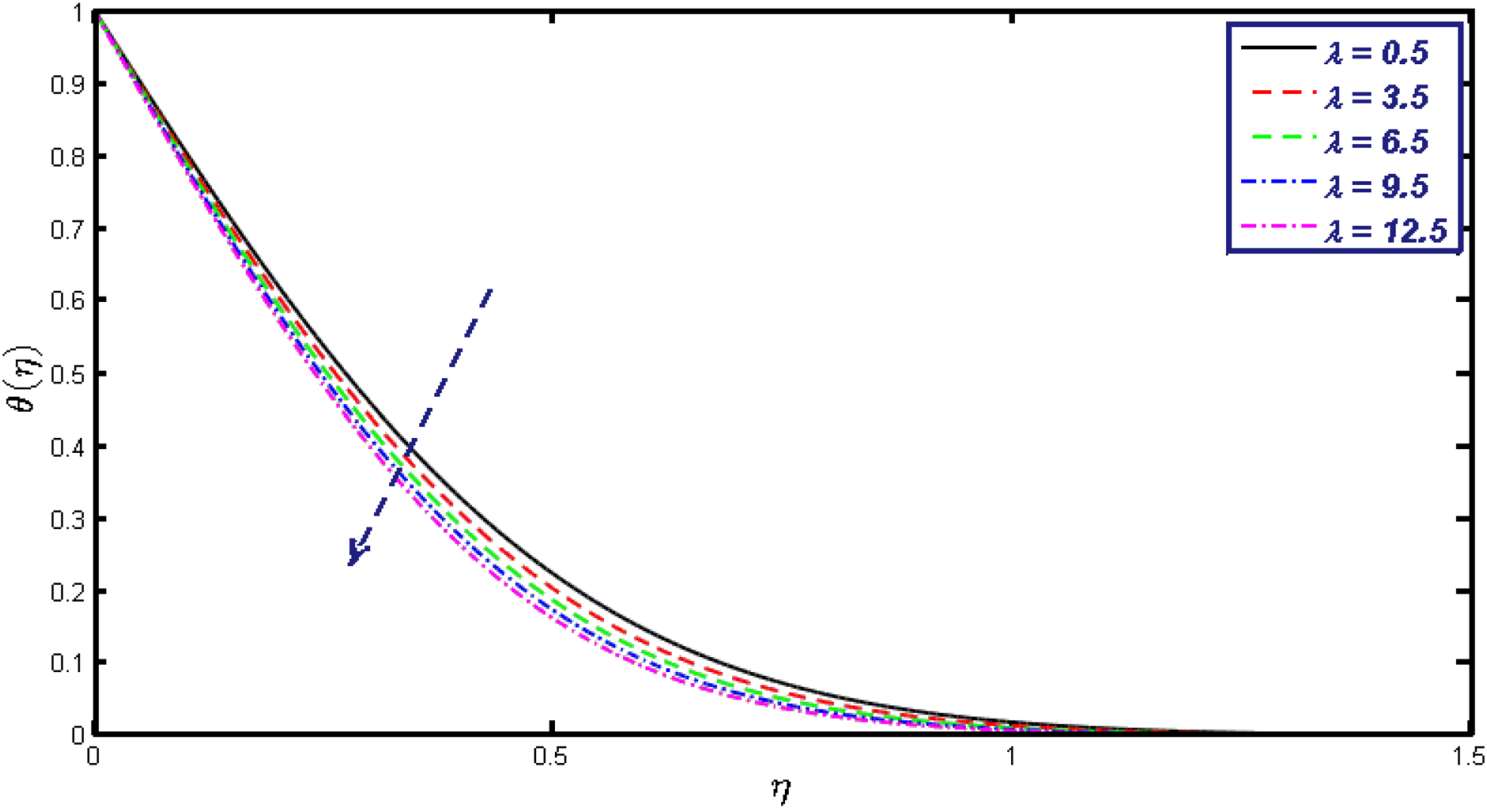
$\theta (\eta )$ field for different values of $\lambda .$

**Figure 11:**
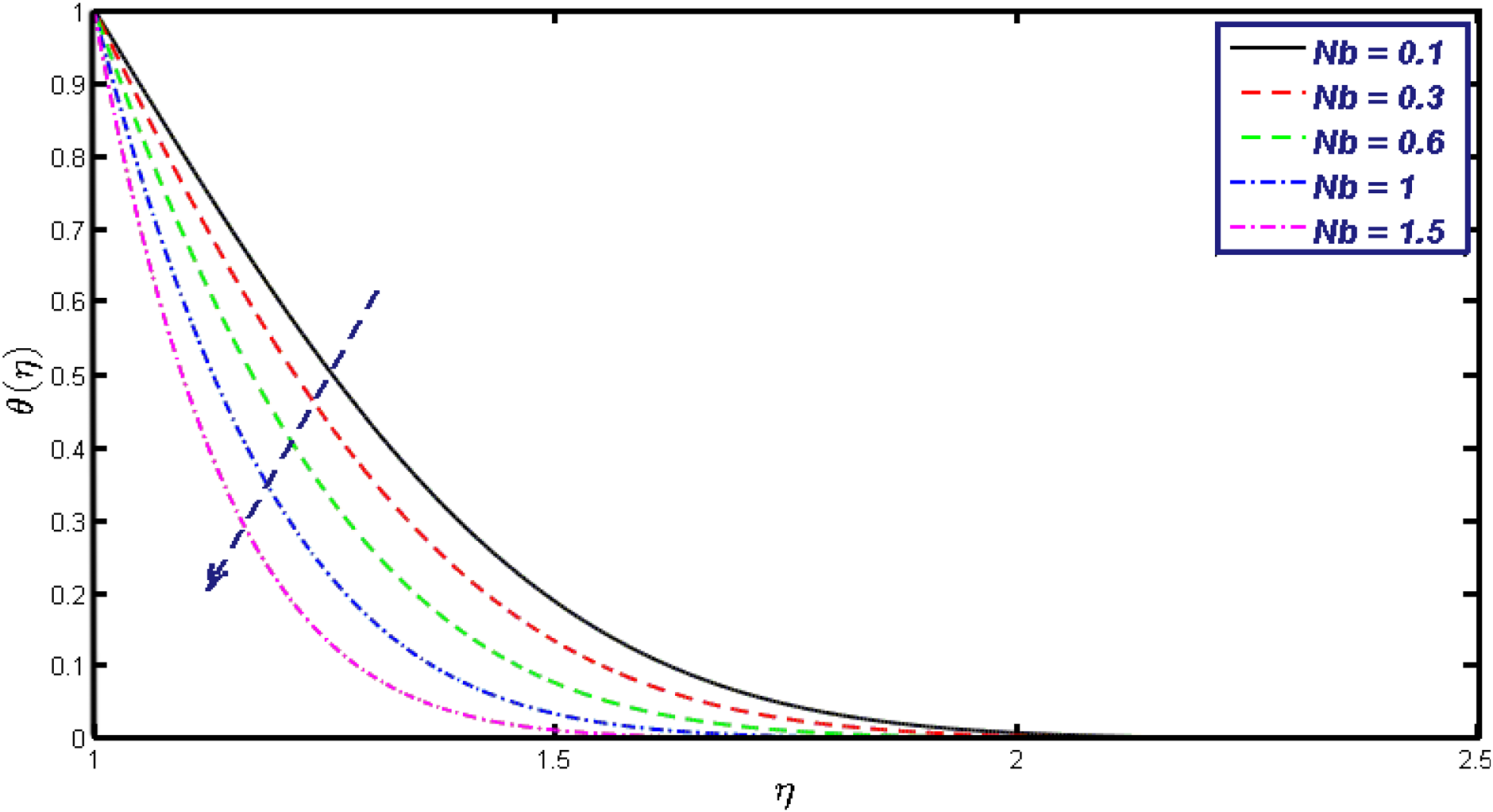
$\theta (\eta )$ field for different values of $Nb.$

**Figure 12:**
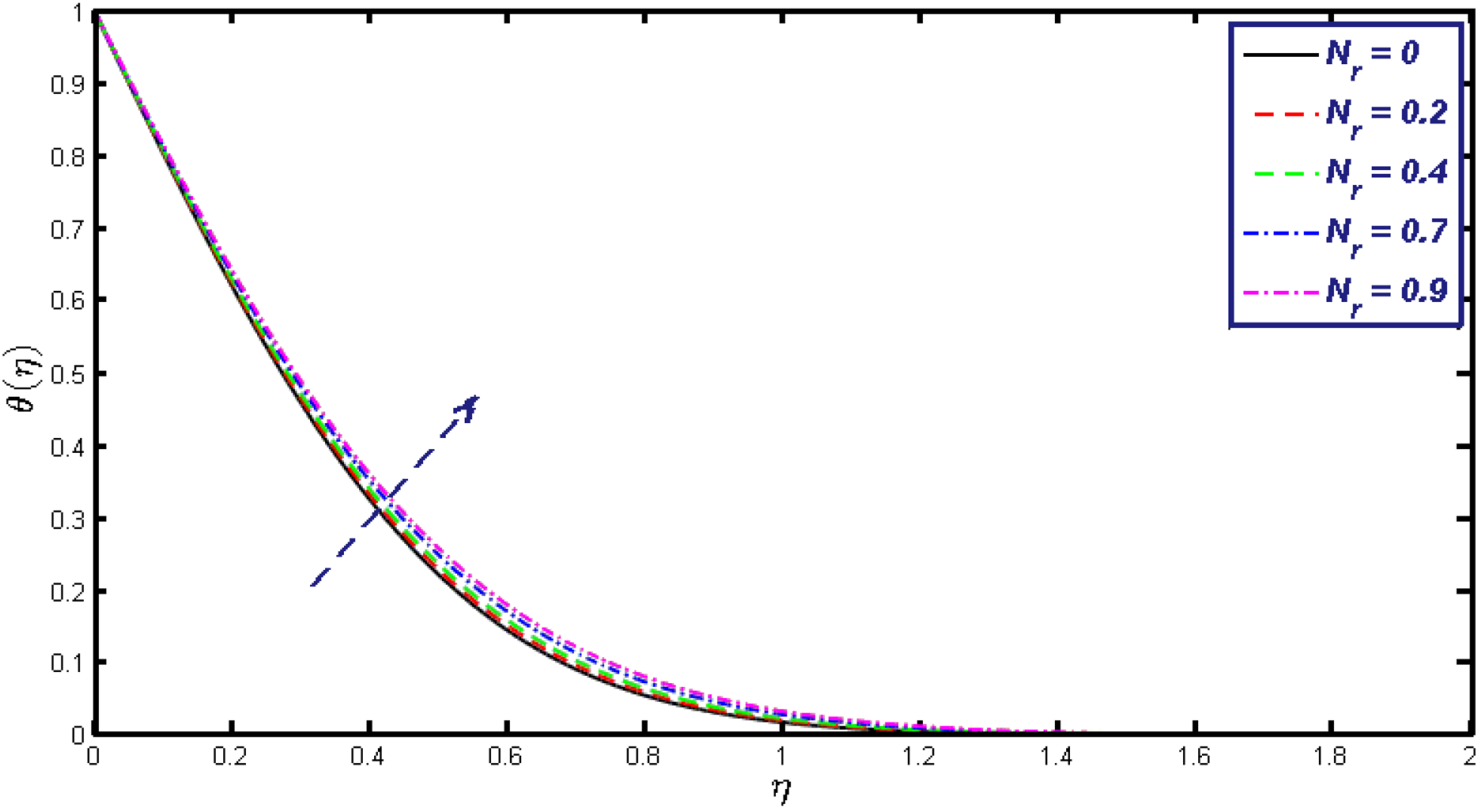
$\theta (\eta )$ field for different values of $Nr.$

**Figure 13:**
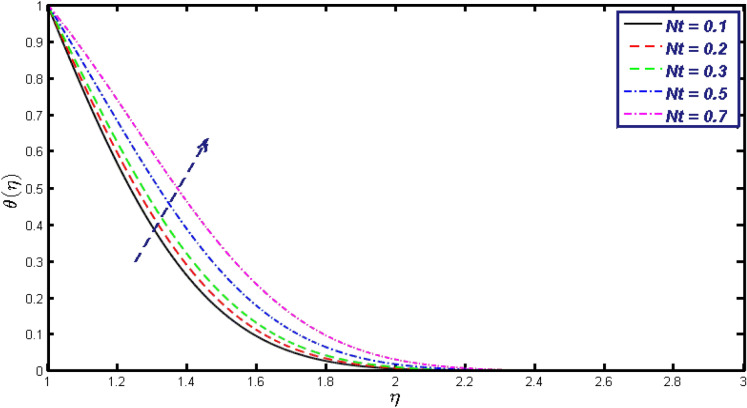
$\theta \left(\eta \right)$ field for different values of $Nt.$

**Figure 14:**
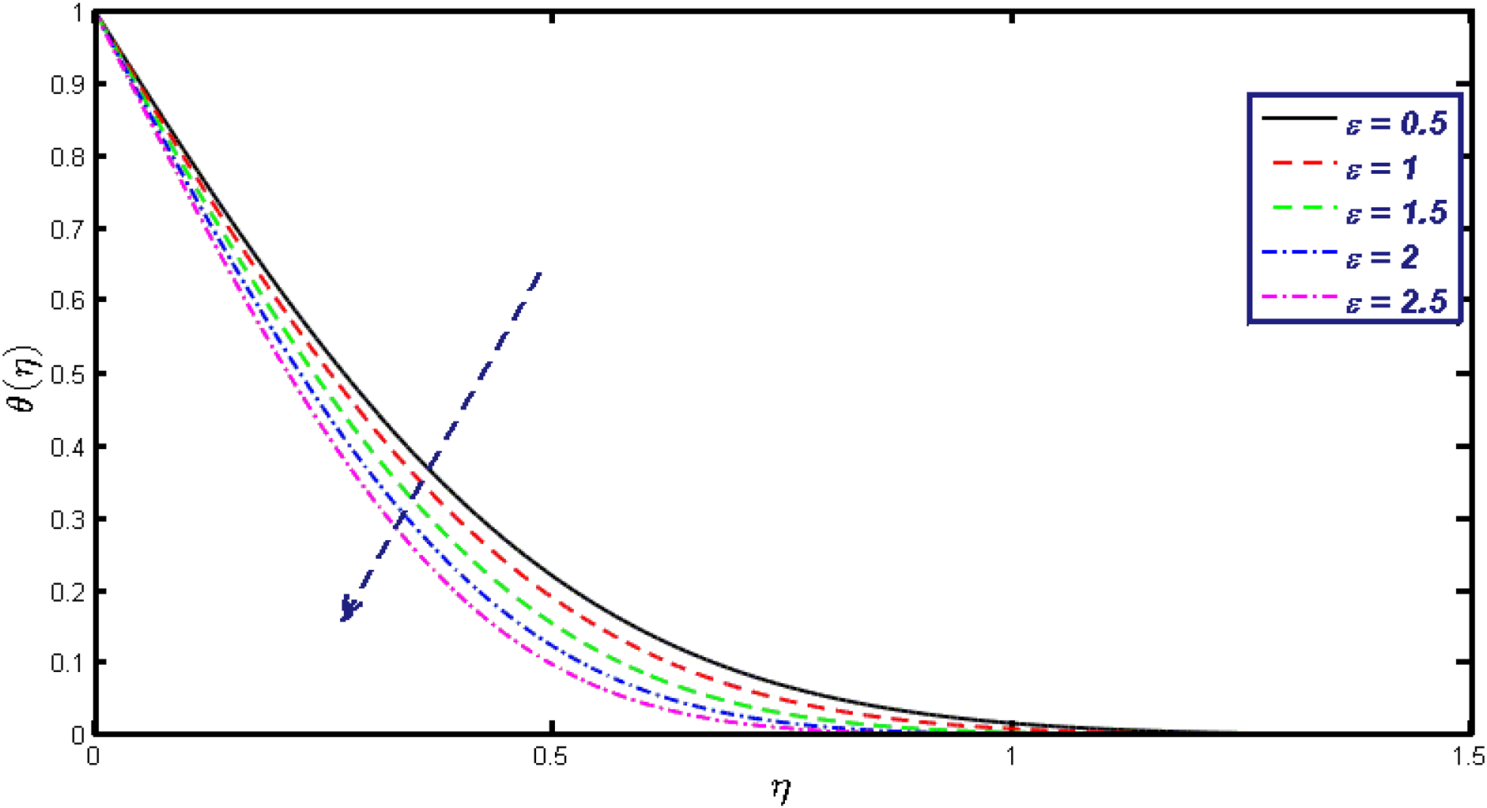
$\theta \left(\eta \right)$ field for different values of $\varepsilon .$

**Figure 15:**
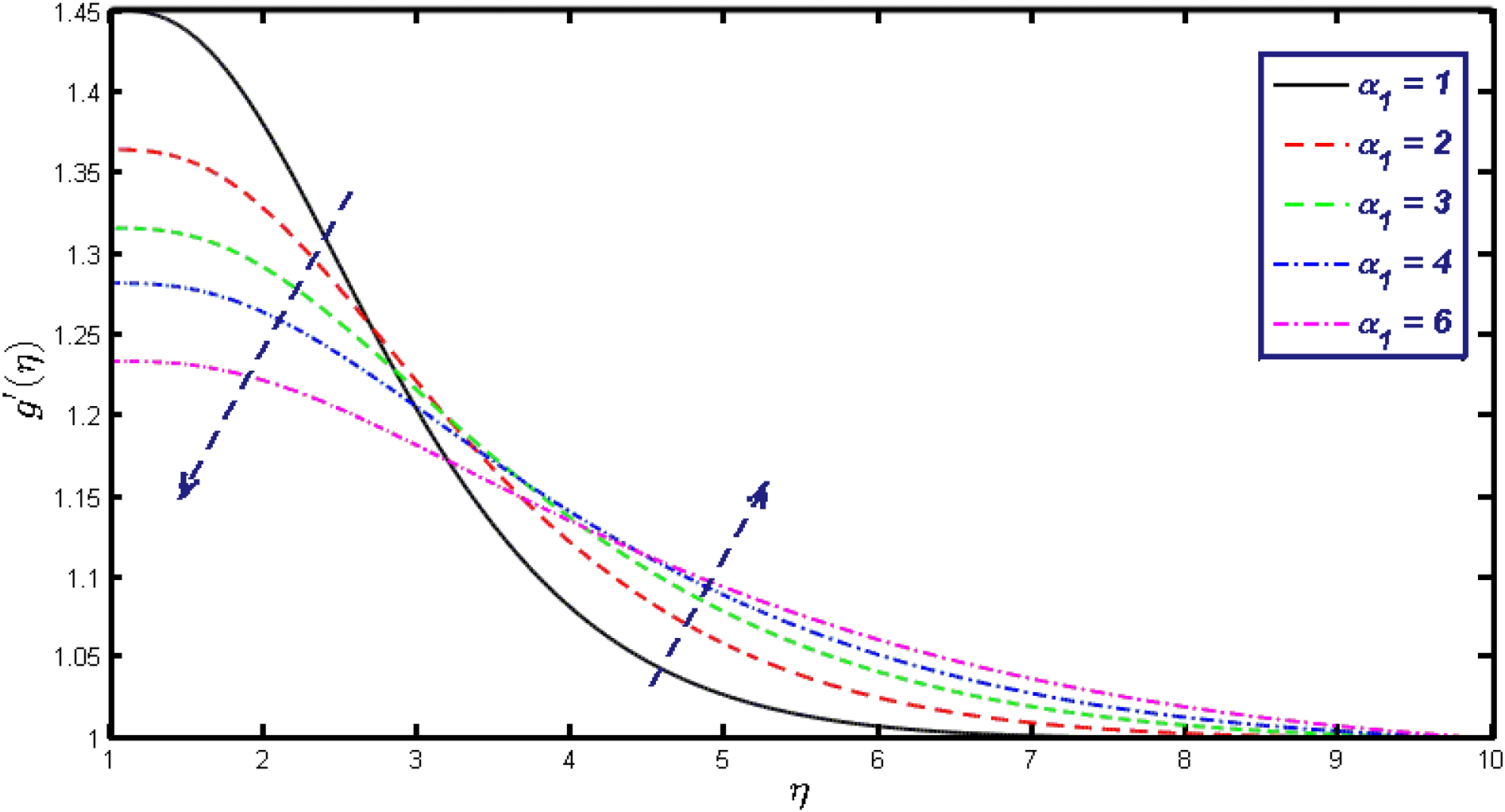
$g{^{\prime}}(\eta )$ field for distinct values of ${\alpha }_{1}.$

**Figure 16:**
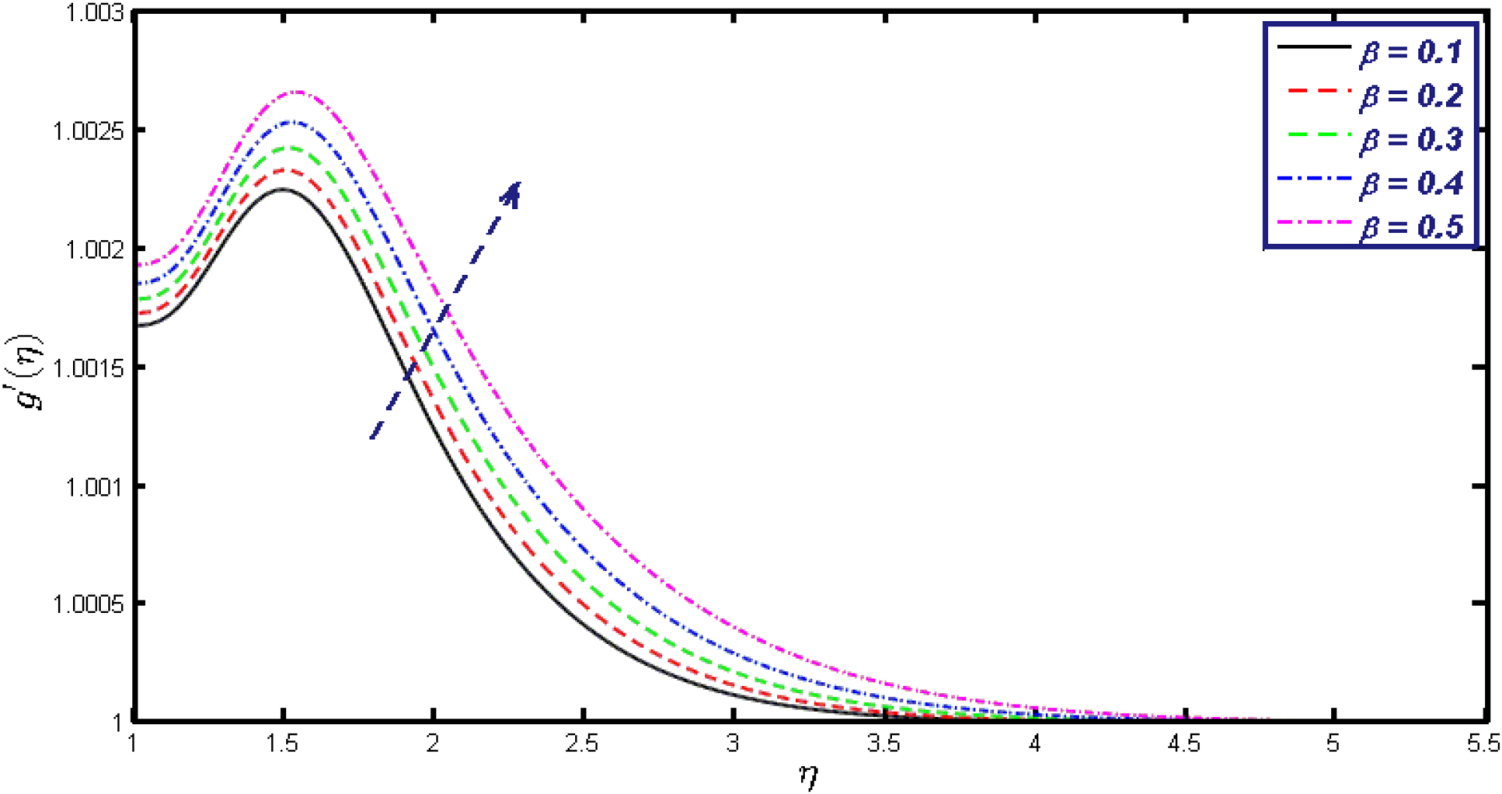
$g{^{\prime}}(\eta )$ field for distinct values of $\beta .$

**Figure 17:**
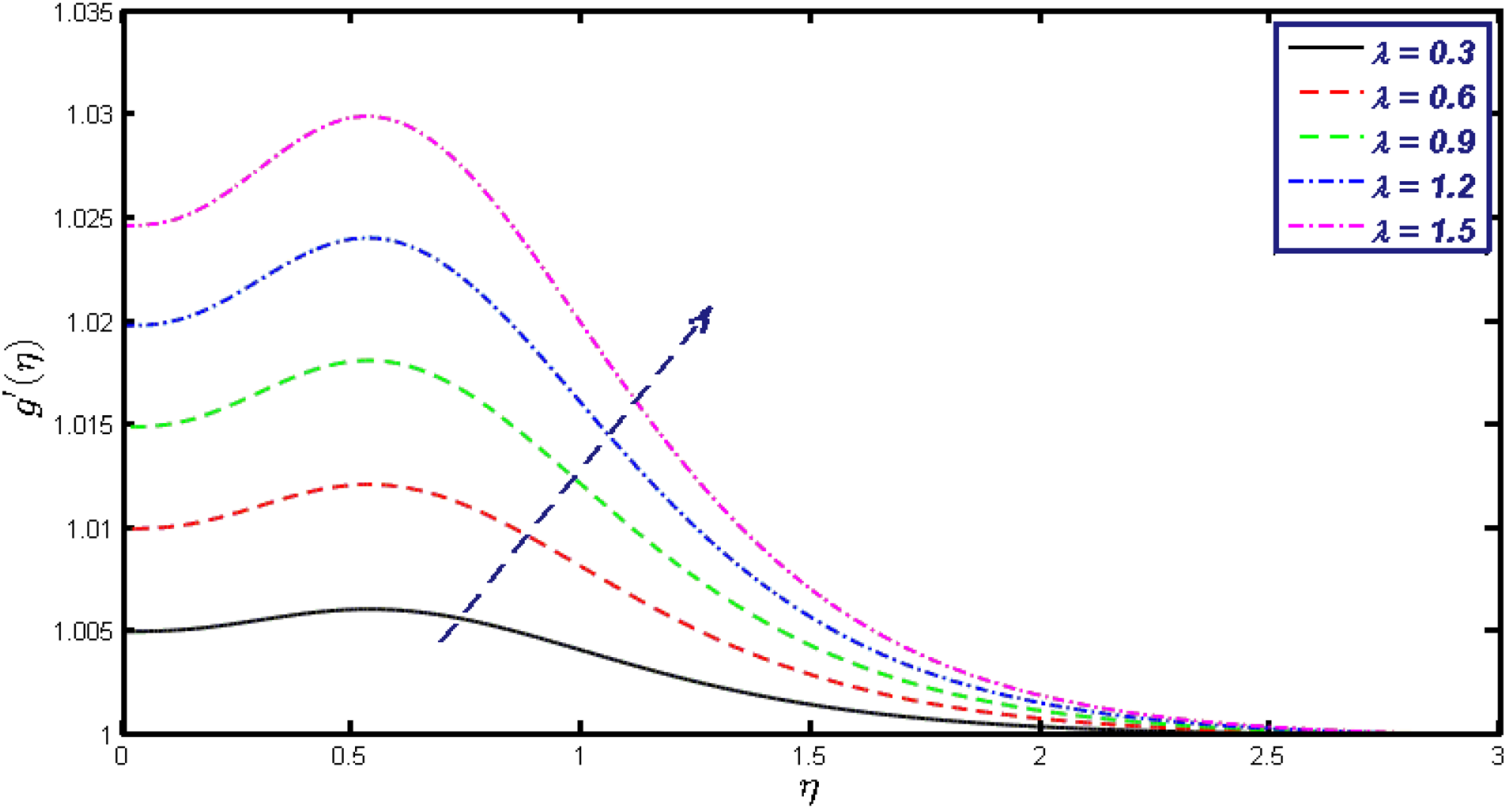
$g{^{\prime}}(\eta )$ field for distinct values of $\lambda .$

**Figure 18:**
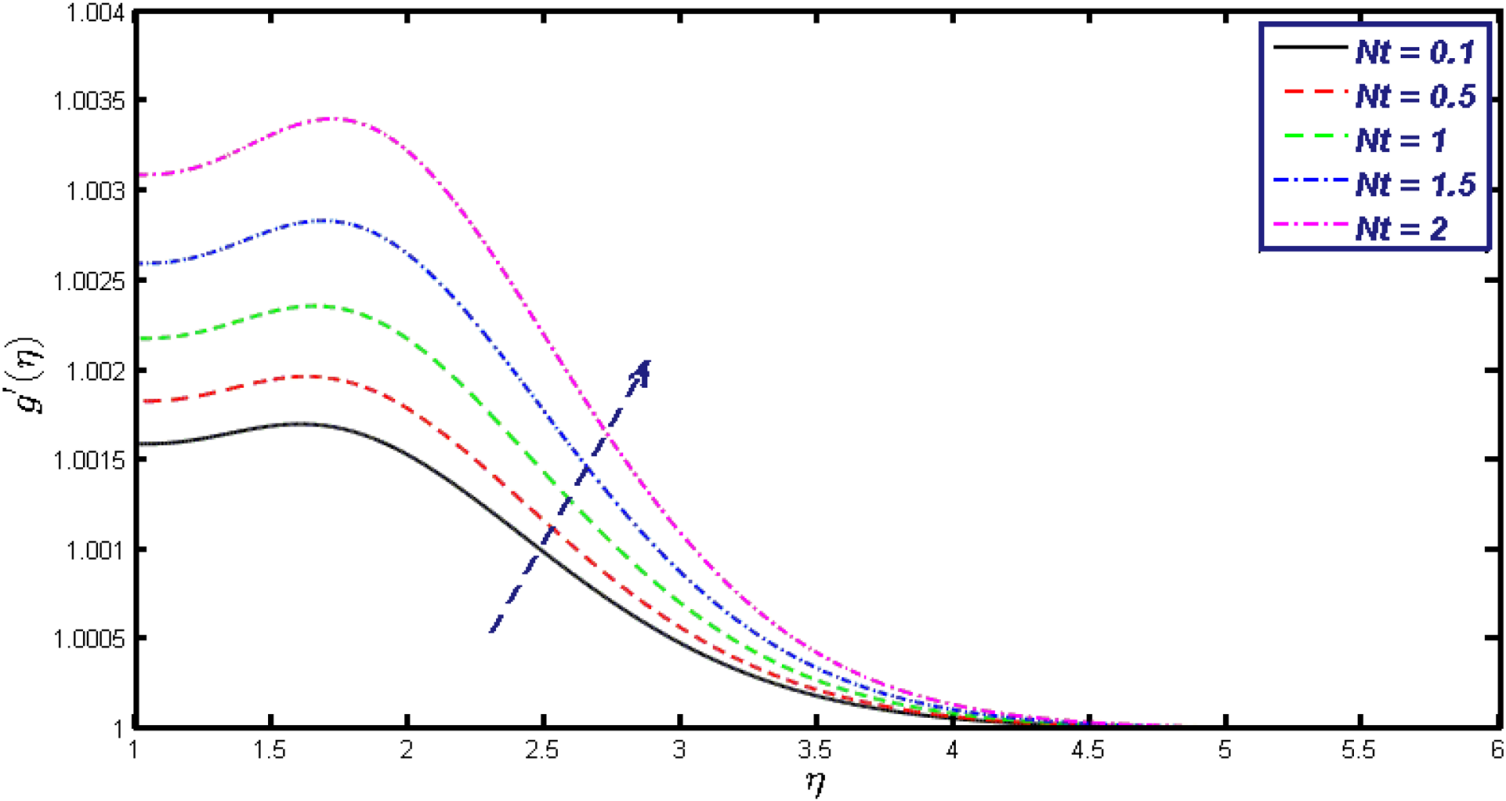
$g{^{\prime}}(\eta )$ field for distinct values of $Nt.$

**Figure 19:**
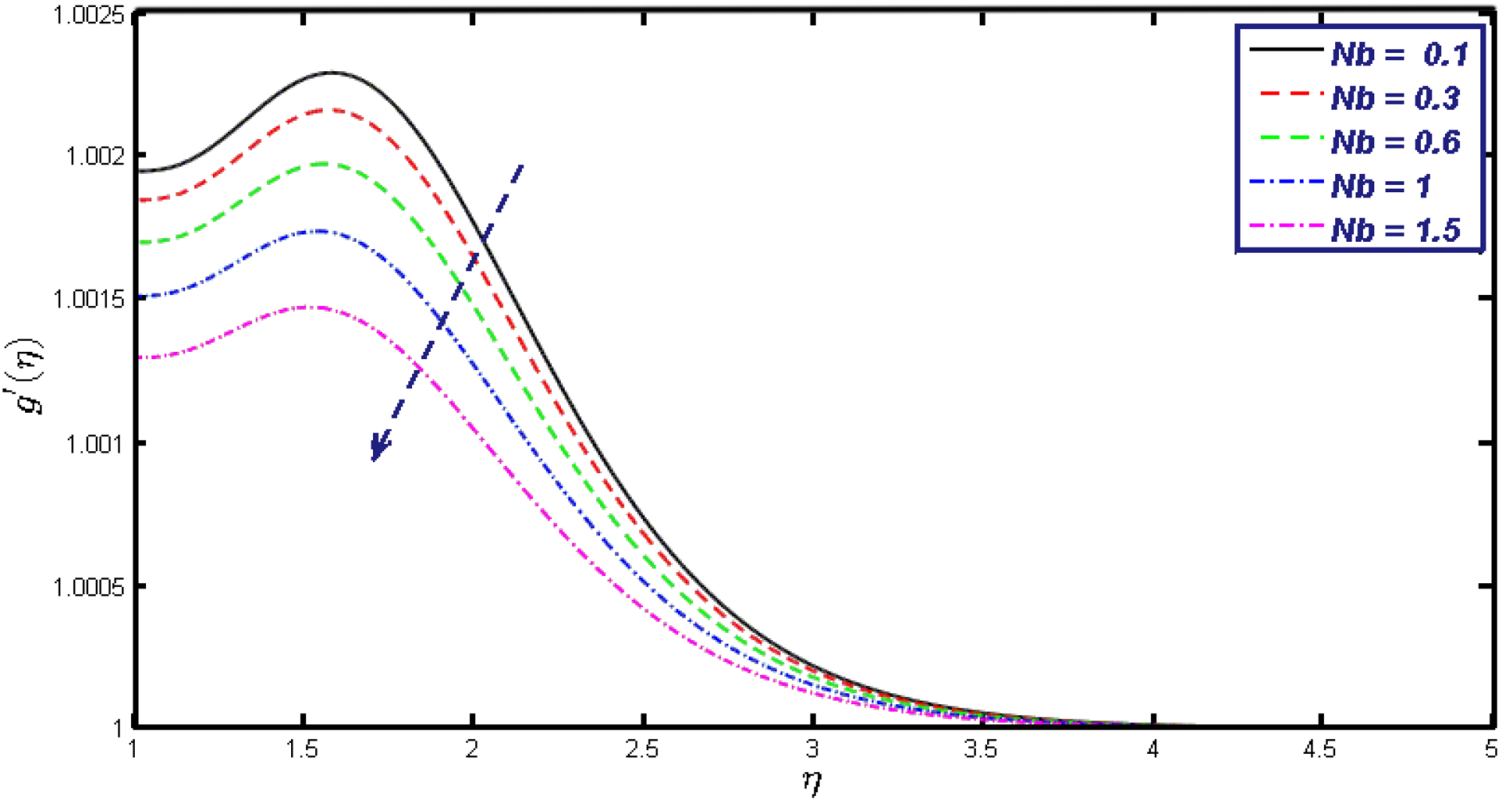
$g{^{\prime}}(\eta )$ field for distinct values of $Nb.$

**Figure 20:**
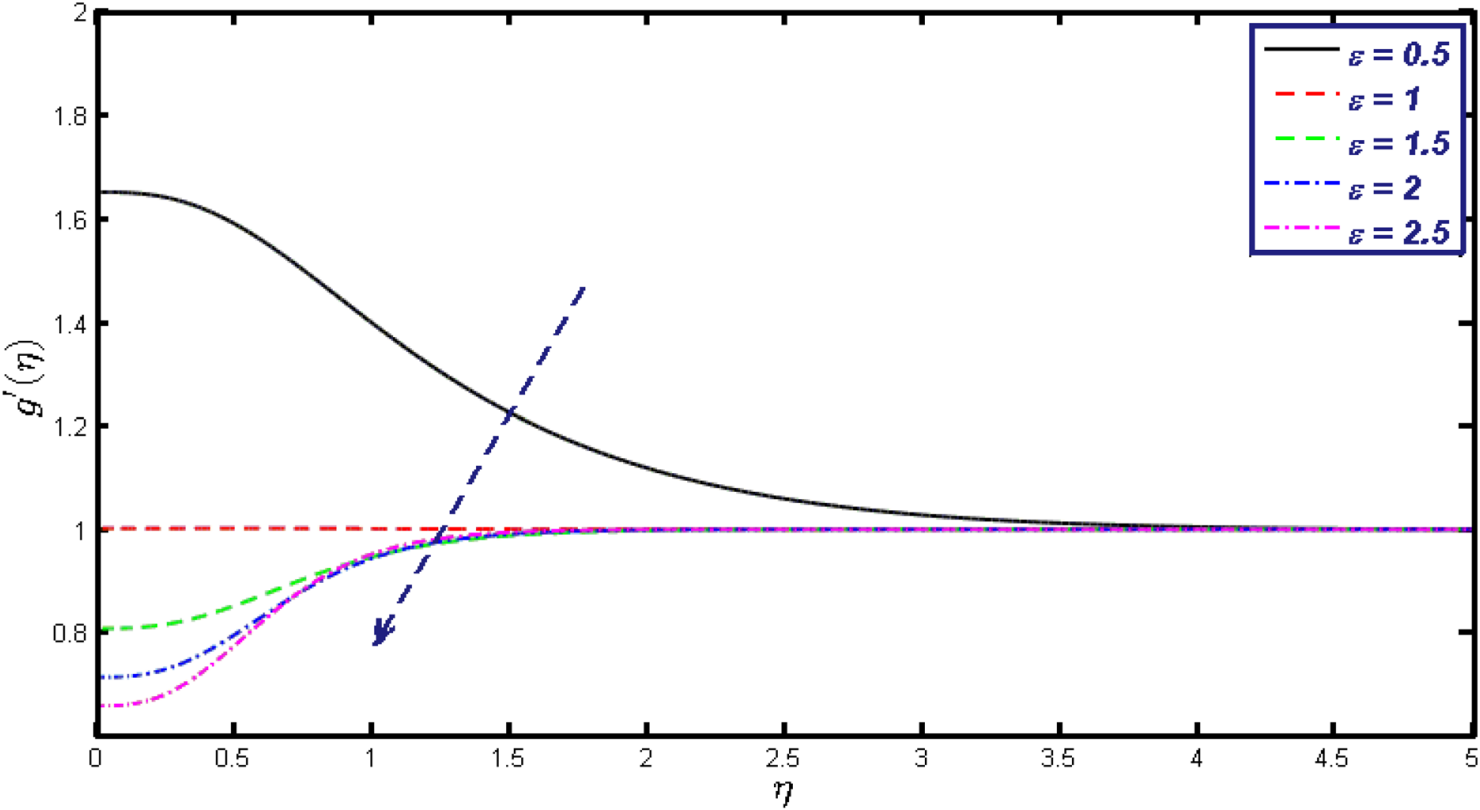
$g{^{\prime}}(\eta )$ field for distinct values of $\varepsilon .$

**Figure 21:**
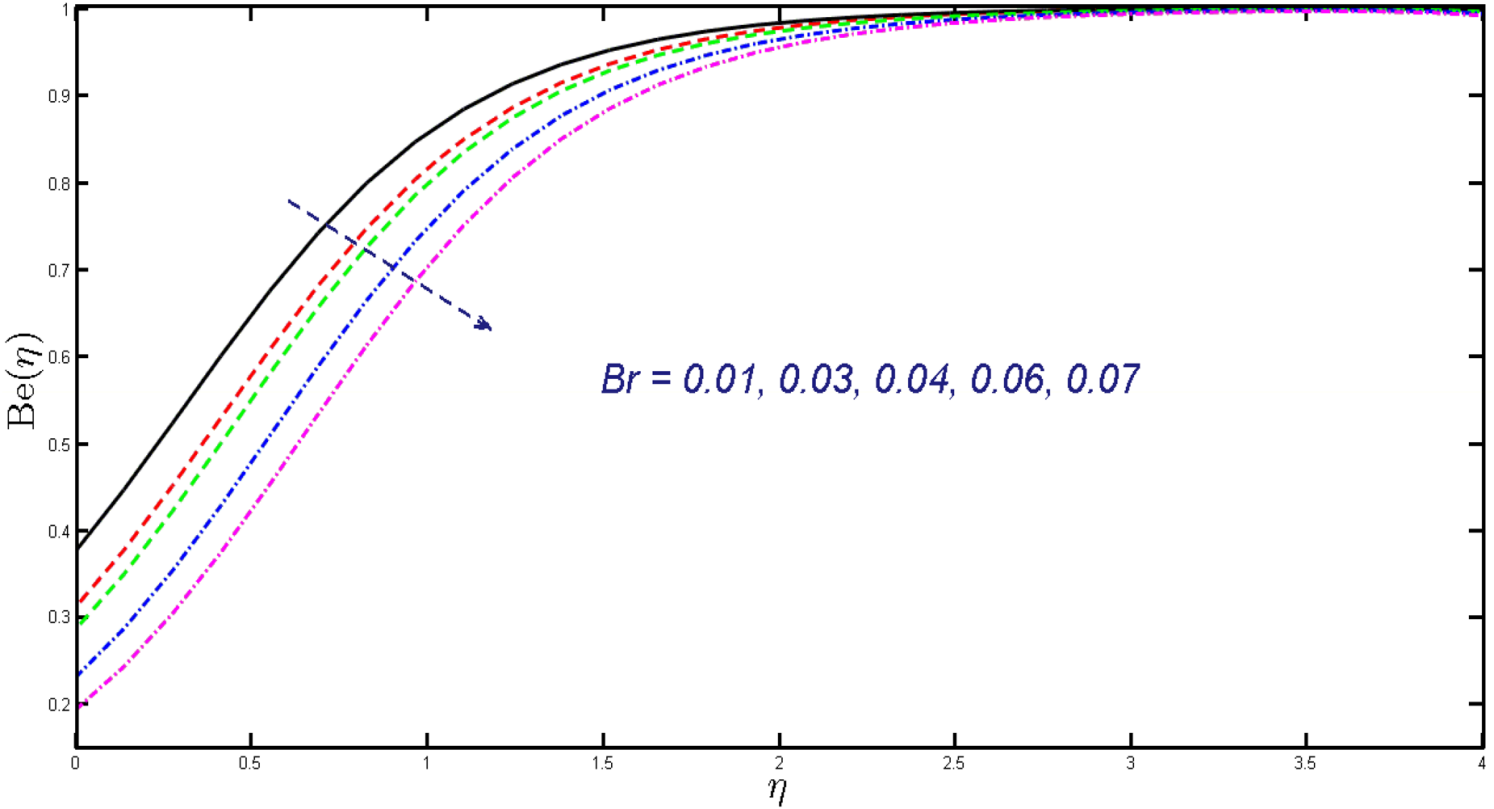
Field of Bejan number for $Br.$

**Figure 22:**
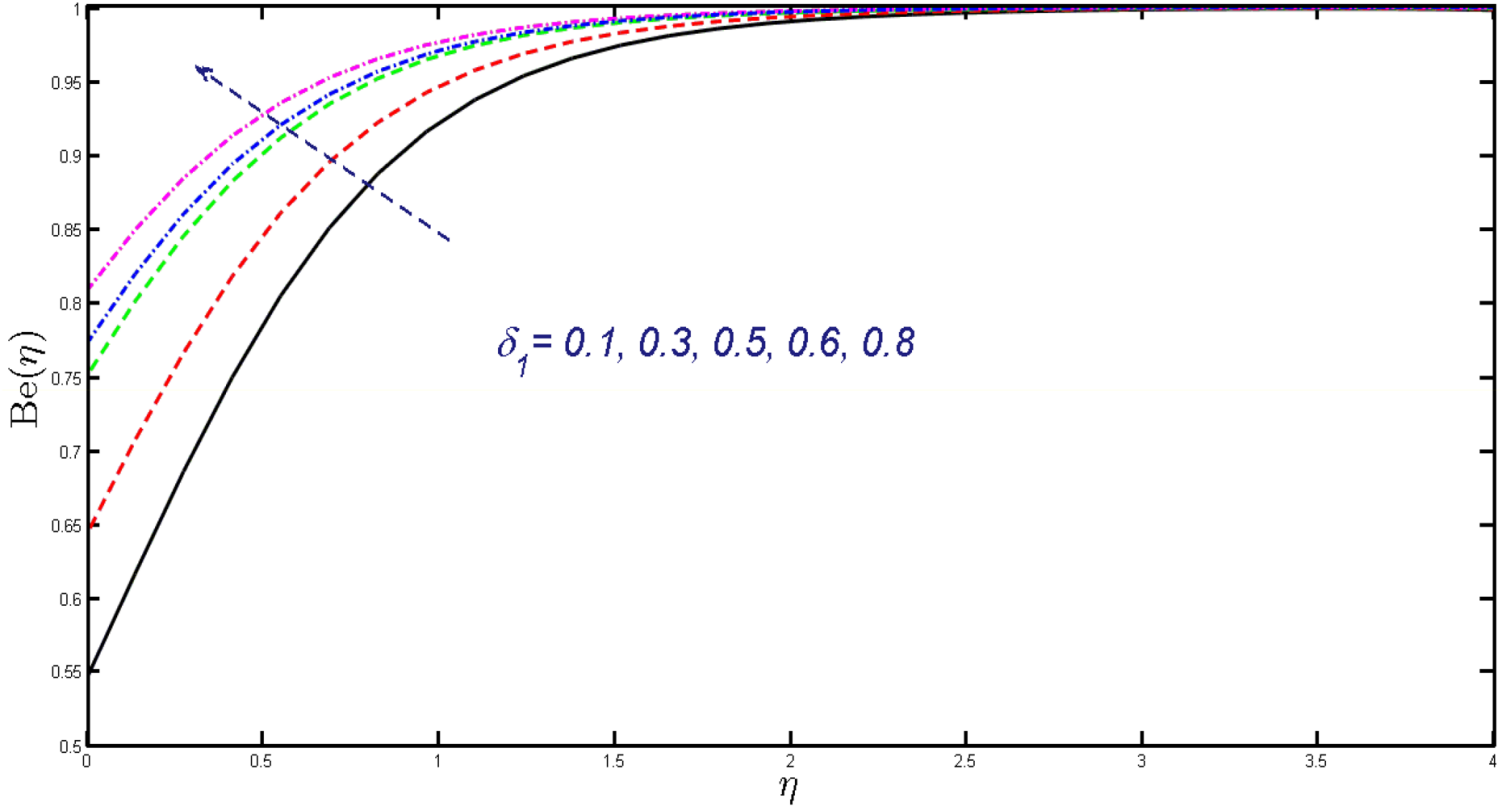
Field of Bejan number for ${\delta }_{1}.$

**Figure 23:**
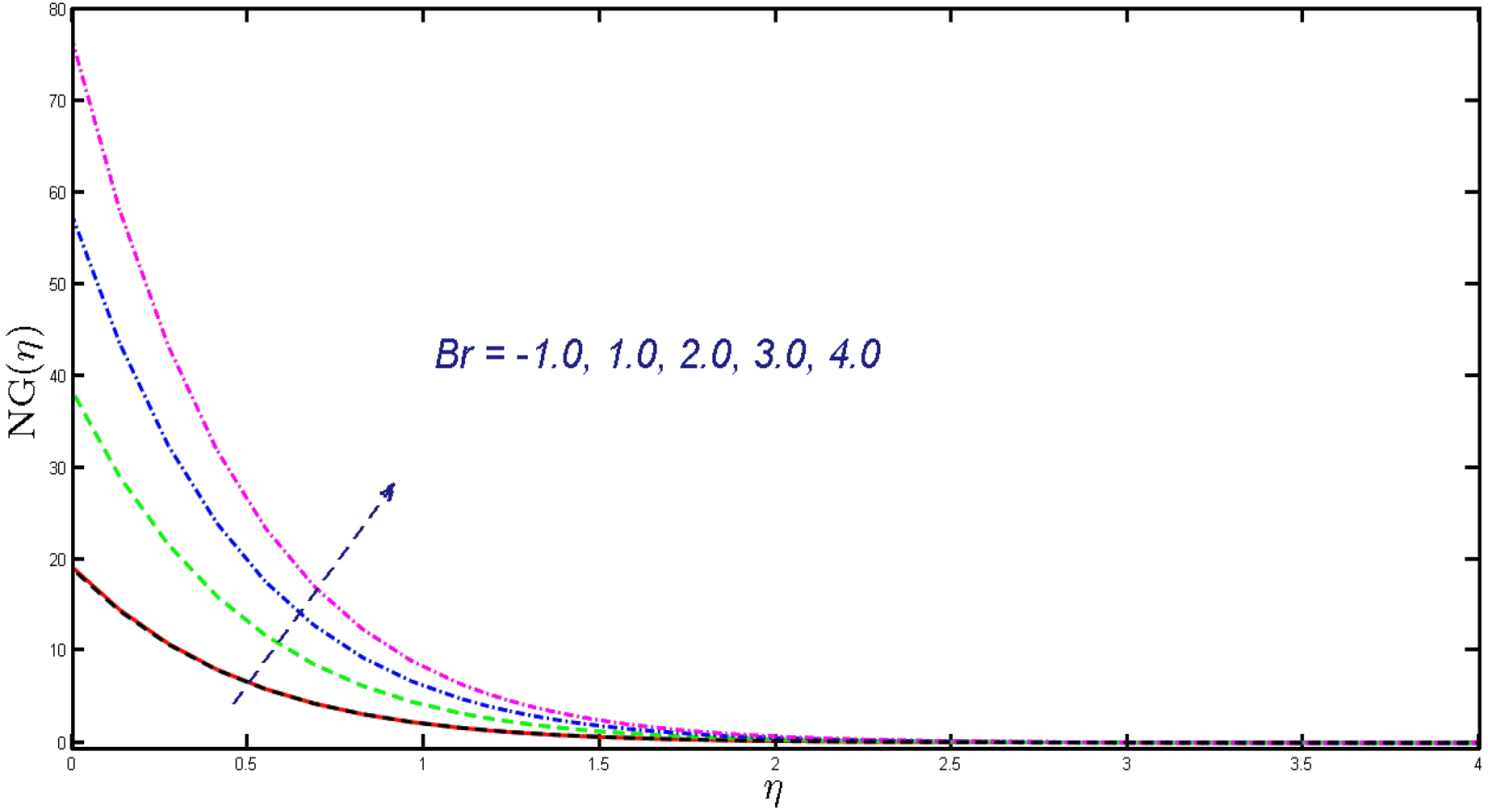
Upshot of $NG(\eta )$ for $Br.$

**Figure 24:**
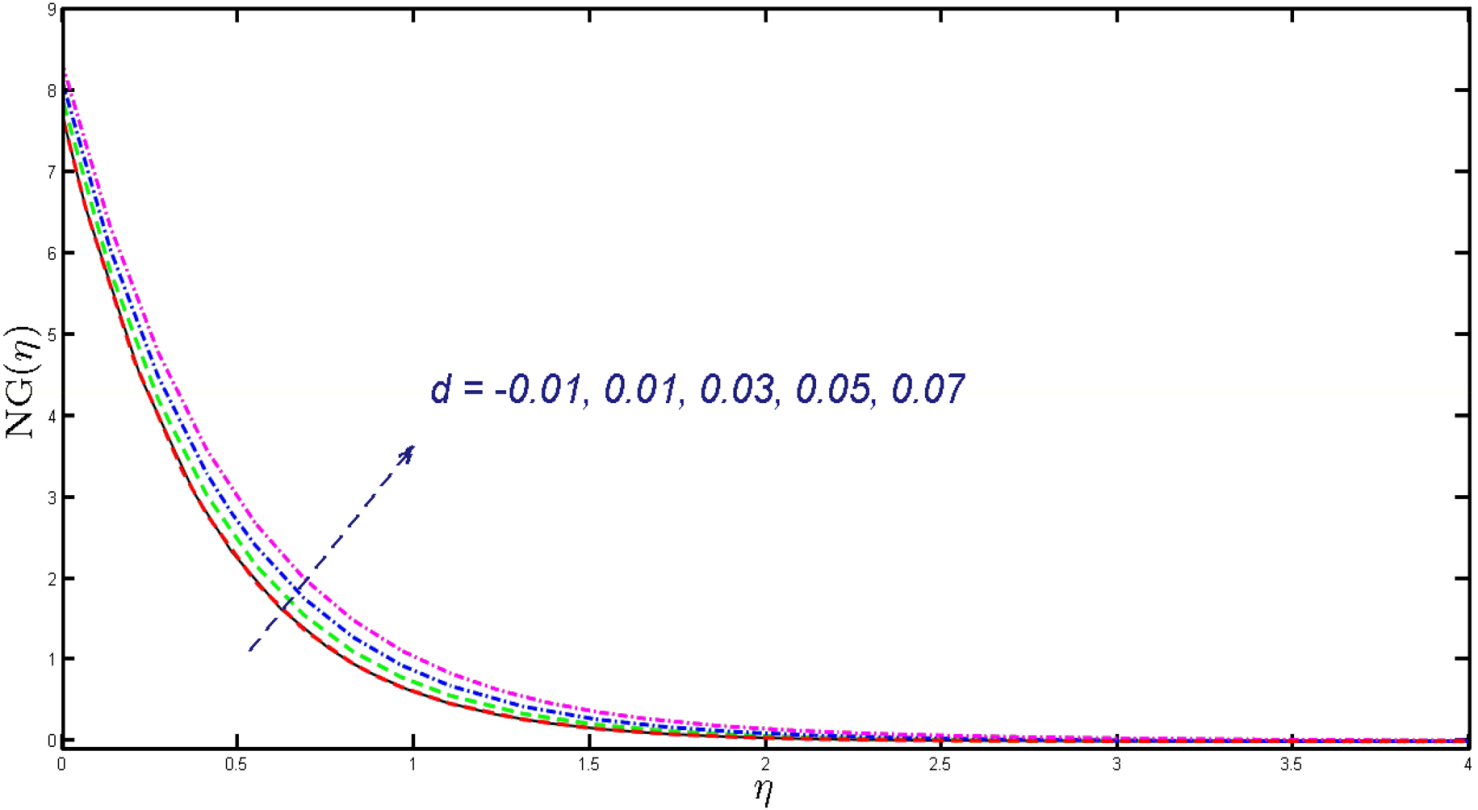
Deviation of $NG(\eta )$ for $d.$

**Figure 25:**
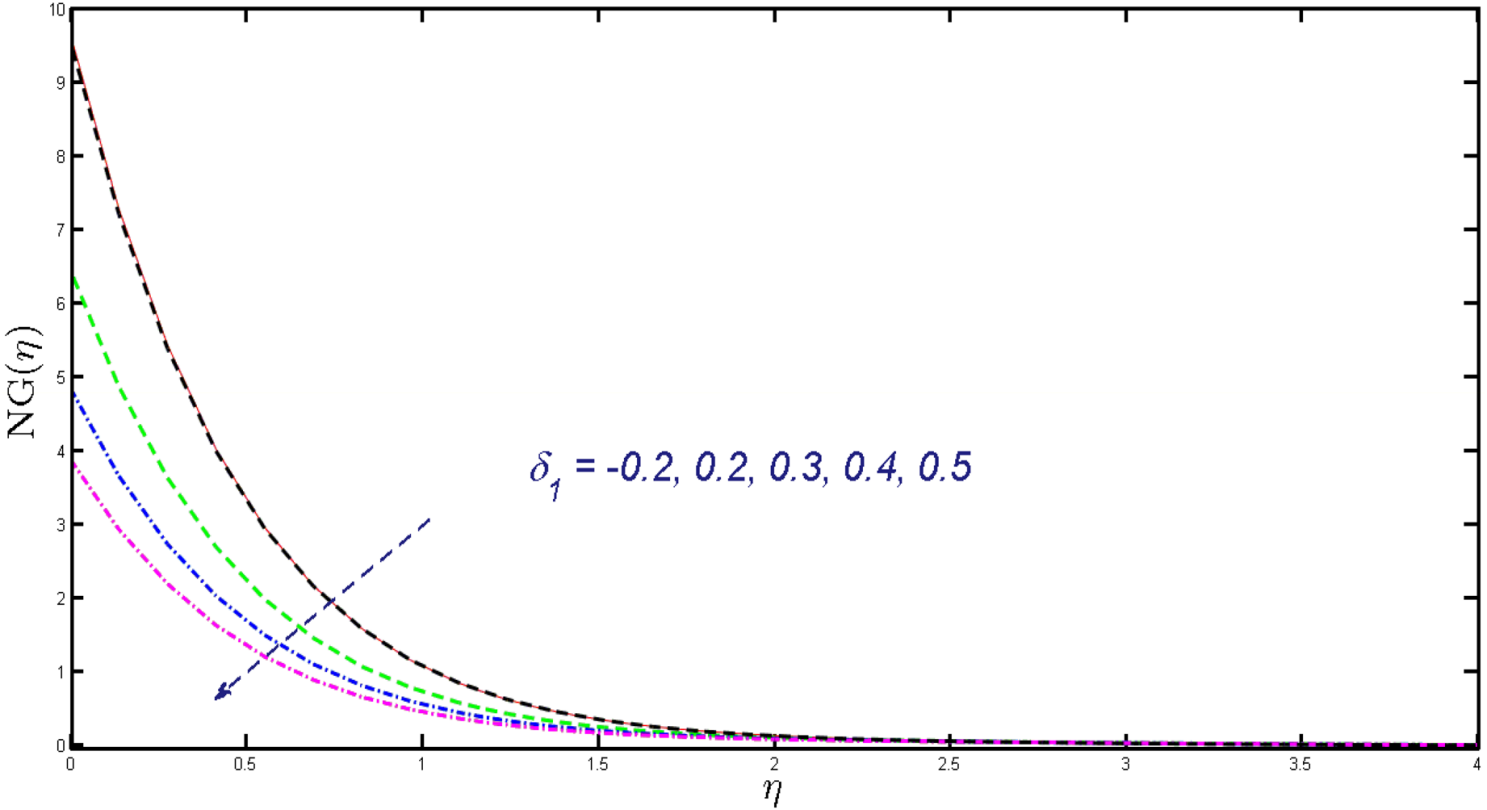
Upshot of $NG\left(\eta \right)$ for ${\delta }_{1}.$

**Figure 26:**
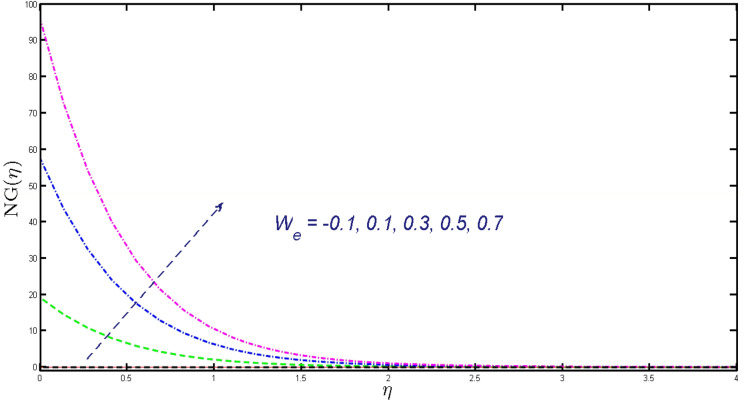
Profile of $NG\left(\eta \right)$ for ${W}_{e}.$

**Figure 27:**
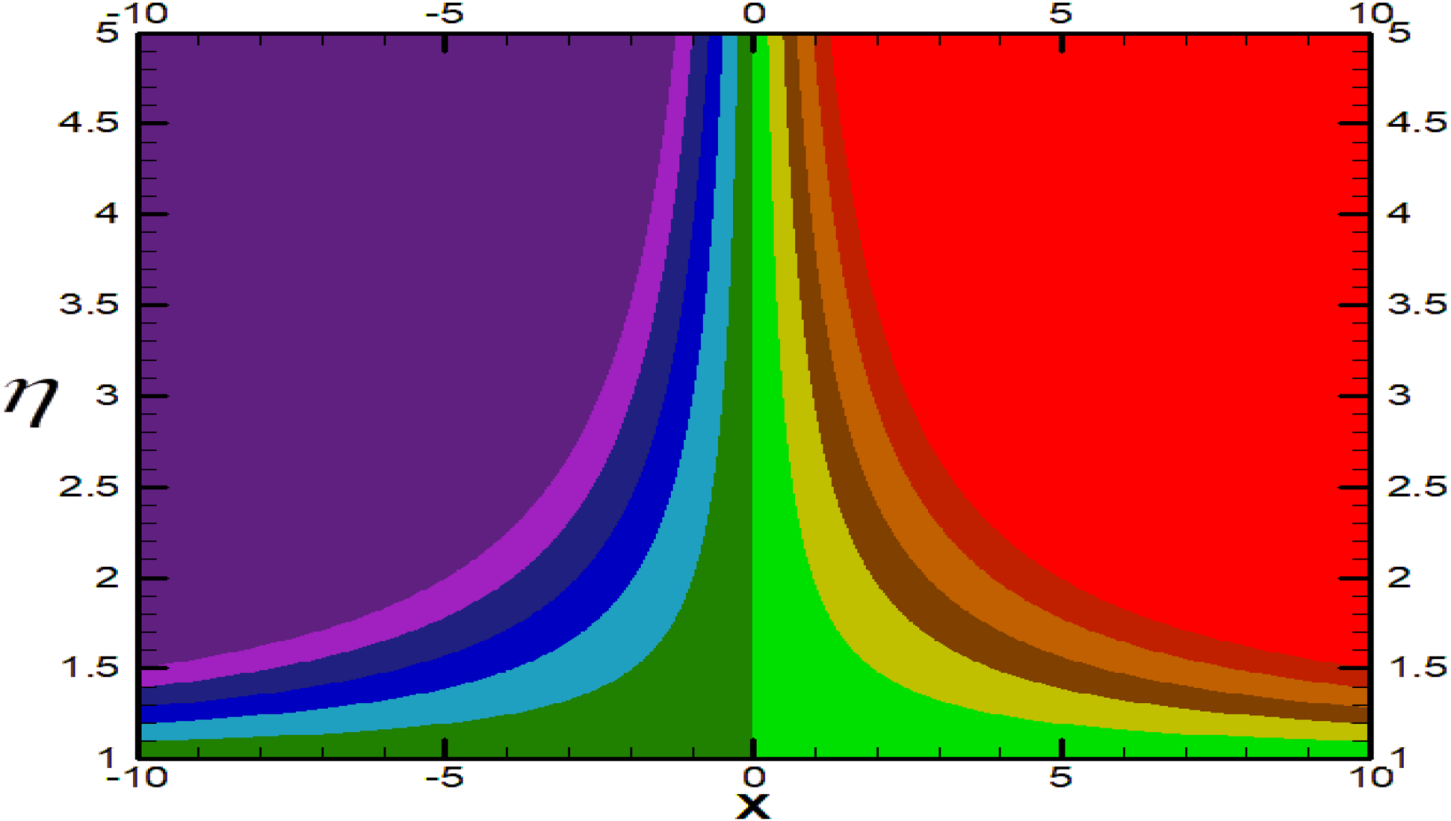
Streamlines for $$\lambda =0.3.$$

**Figure 28:**
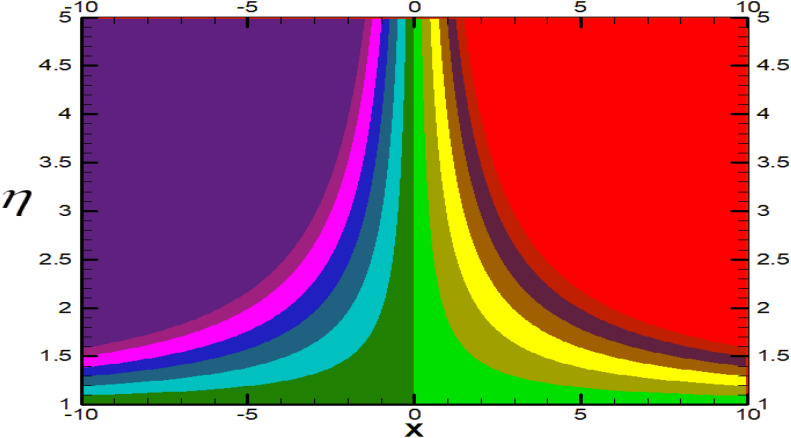
Streamlines for $$\lambda =0.9.$$

**Figure 29:**
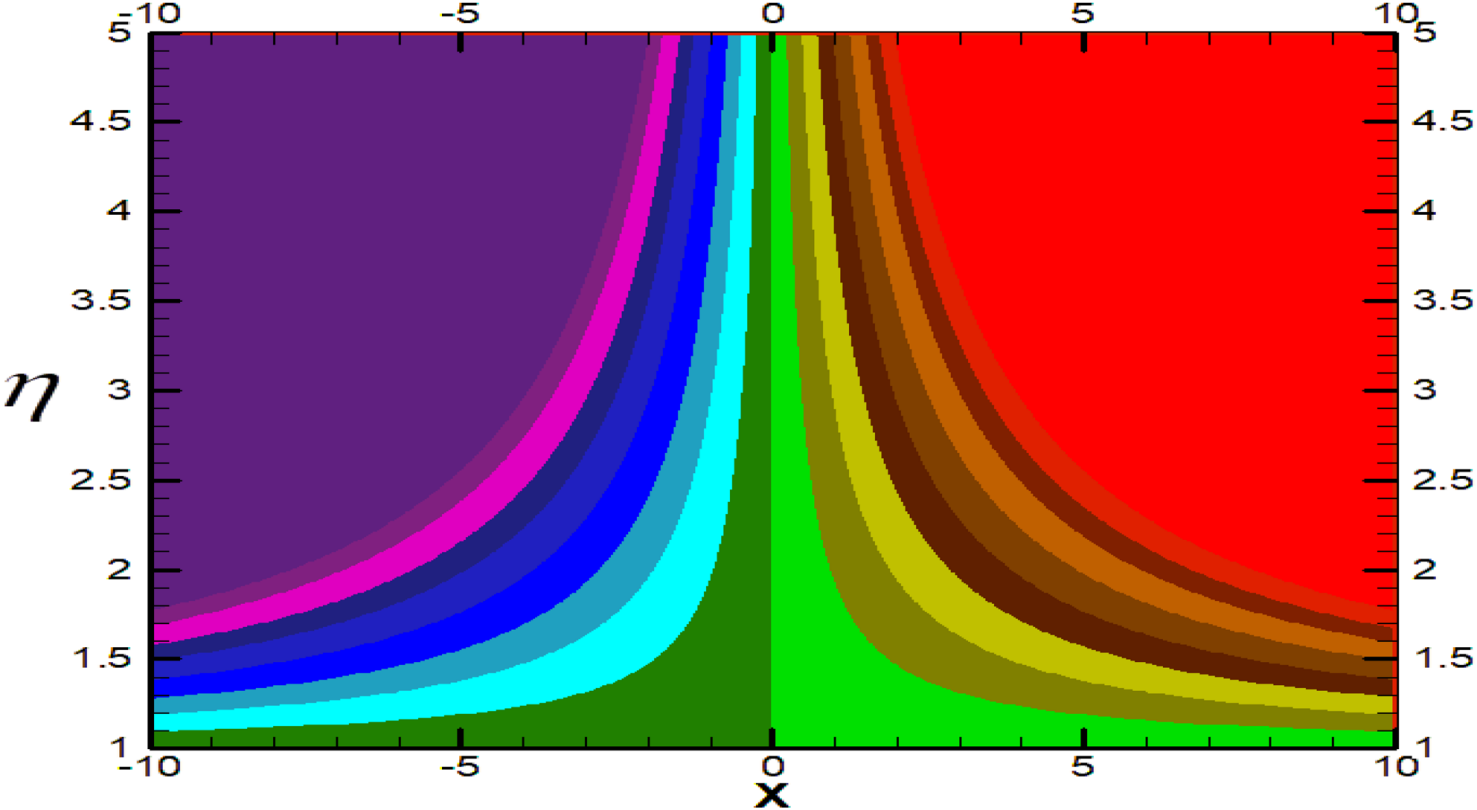
Streamlines for $$\lambda =1.5.$$

**Figure 30:**
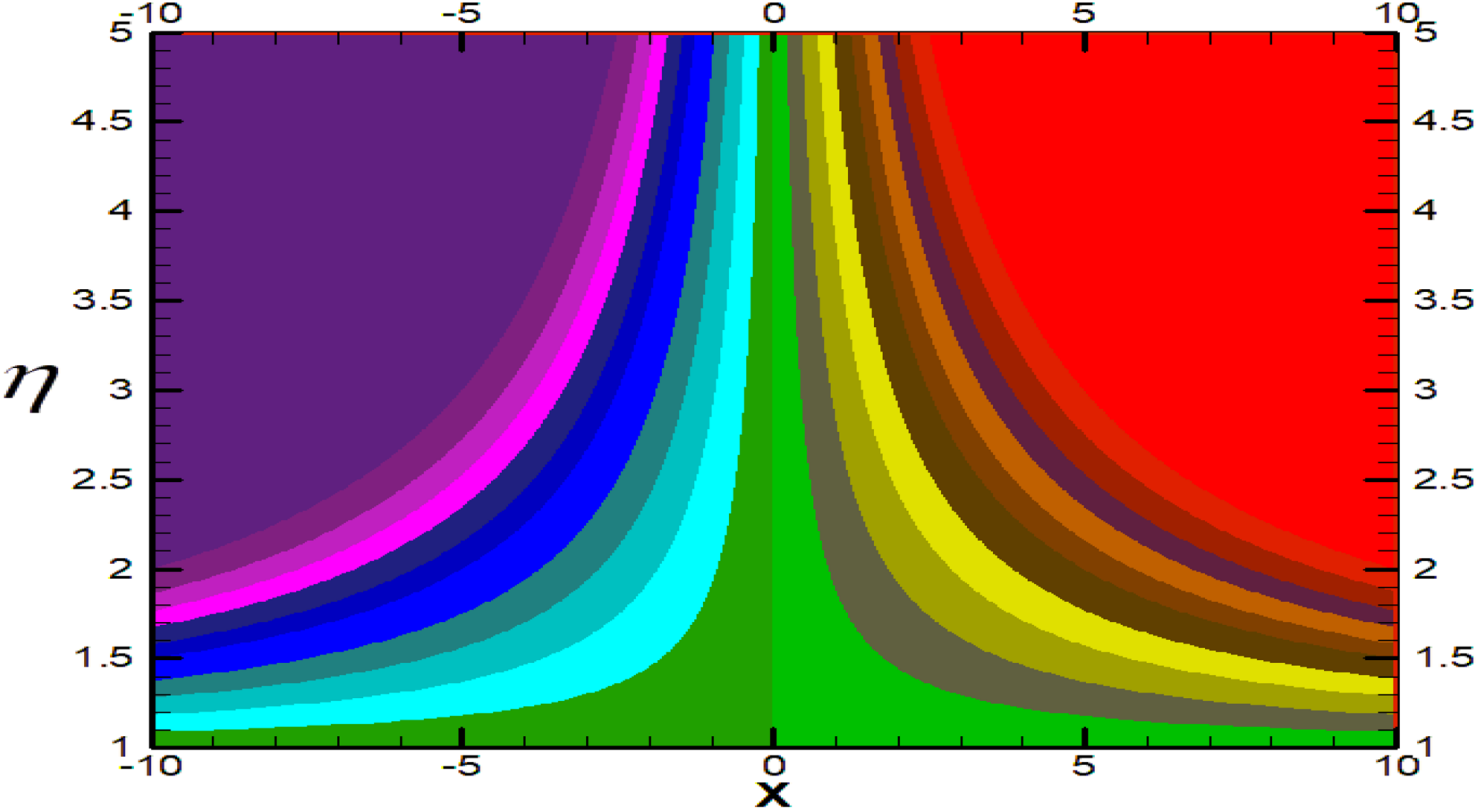
Streamlines for $$\beta =0.1.$$

**Figure 31:**
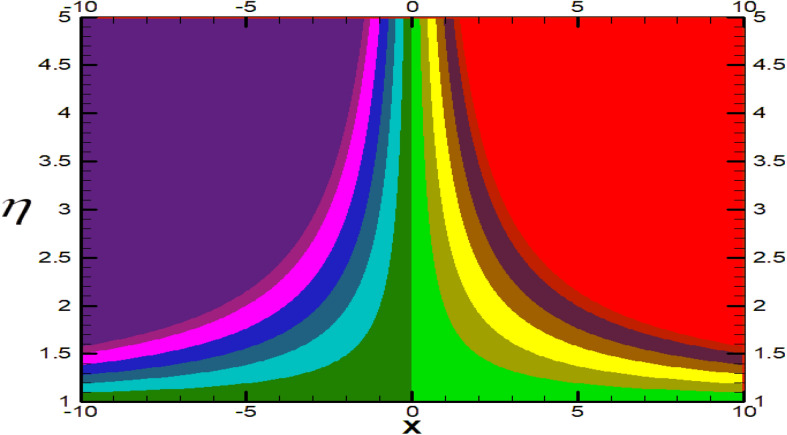
Streamlines for $$\beta =0.3.$$

**Figure 32:**
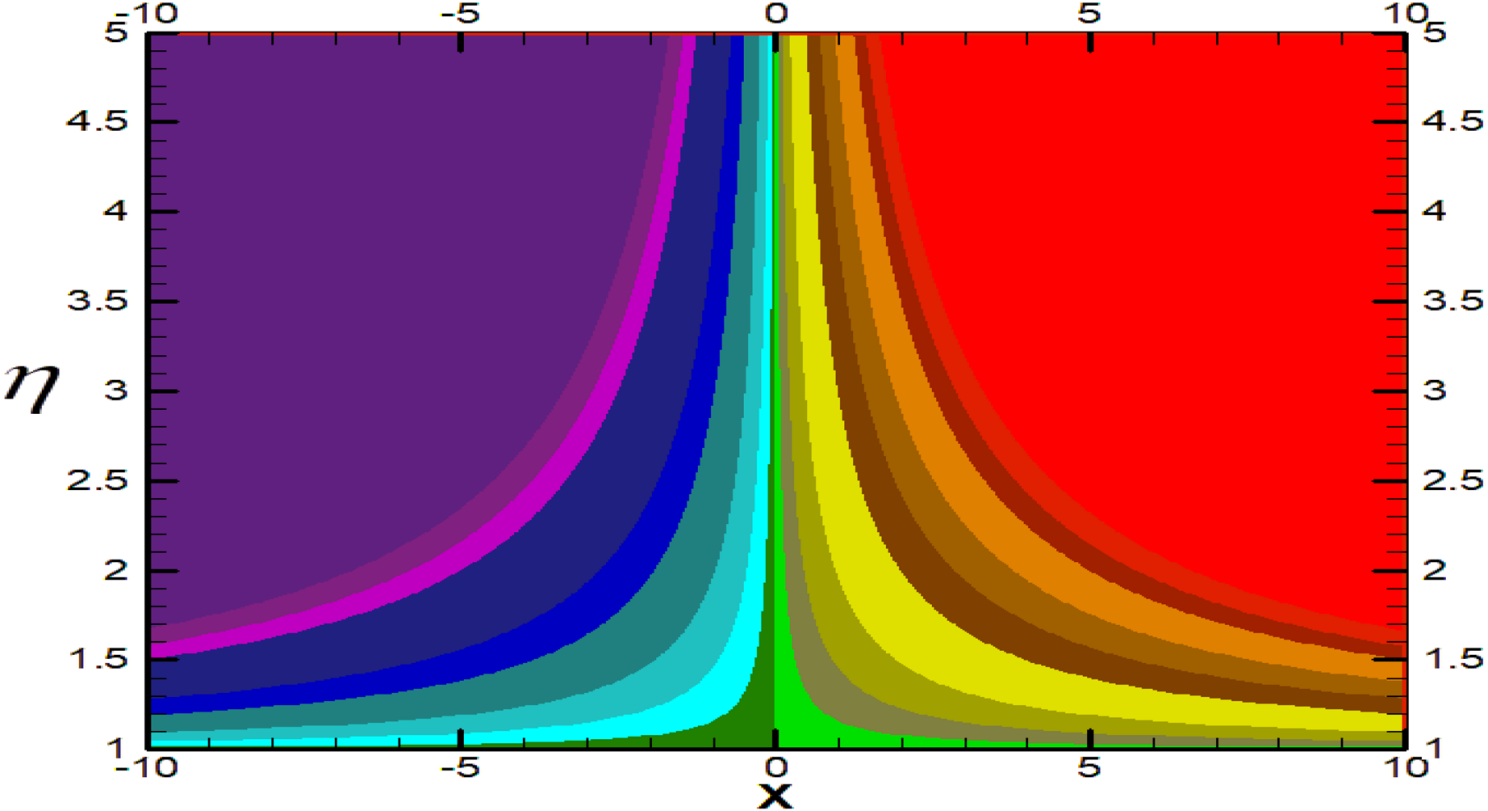
Streamlines for $$\beta =0.5.$$

Non-linear differential Eqs. (13–16) with boundary conditions (17, 18) are worked out by applying the bvp4c MATLAB algorithm. In this section graphical consequences of the numerical solution are clarified to compare the impacts of distinct values of parameters on flow characteristics. Figure 2 demonstrates the effects of ${\alpha }_{1}$ on velocity distribution, curve decays with increased values of ${\alpha }_{1}$. Figure 3 describes the effect of induced magnetic parameters on the field of velocity. It is noticeable that the curve grows when the value of $\beta$ increases. Usually, an increase in the induced magnetic field develops the electric current. This electric force can help to enhance the momentum boundary layer thickness. This leads to an increase in the momentum boundary layer thickness. In Fig. 4 it is clear that when the mixed convection parameter rises the velocity profile moves upward. Figure 5 discussed the impact of $Nb$ on the pattern of velocity. Momentum boundary layer thickness goes down when the values of $Nb$ inclined. Figure 6 defines the impact of $Nt$ on the velocity curve. The graph of $Nt$ raises when the values of $Nt$ increase. Figure 7 describes the impact of stretching parameter on the velocity field, improves in the value of $\varepsilon$ field of velocity exceed. An increase in the stretching parameter initially develops more pressure on the flow; due to this reason, we have seen an enhancement in the velocity profiles. Figure 8 represents the temperature field of ${\alpha }_{1}$, the field of heat enlarge when the value of ${\alpha }_{1}$ improved. Figure 9 temperature profile of $\beta$ goes down when the value of $\beta$ enhanced. Figure 10 shows that on temperature distribution curve went down by inclining the value of $\lambda$. Figure 11 scrutinizes the impact of $Nb$ on temperature field, heat profile goes down while inclining the values of $Nb$. Figures 12 and 13 describes the consequences of ${N}_{r}$ and $Nt$ on heat transfer distribution, profile decrease while enlarging the values of these parameters. It can be easily noticeable that Fig. 14 shows the impact of the stretching parameter on temperature profile, the heat transfer field goes down when we expand the values of the stretching parameter. Figure 15 explored the impacts of reciprocal of the magnetic Prandtl number on the induced magnetic field, ${\alpha }_{1}$ field decreases near the boundary and increases far away with inclining amount of ${\alpha }_{1}$. Induced magnetic field upgrade while increasing the amount of the magnetic parameter in Fig. 16. Physically Lorentz force decreases through a higher amount of magnetic function. Mixed convection and thermophoresis parameter have been defined same behavior on the induced magnetic curve, profile increased by rising these parameters in Figs. 17 and 18 respectively. Induced magnetic profile getting down when we rising the amount of nanofluid Brownian parameter and stretching parameter in Figs. 19 and 20. Physically Lorentz force increases through bigger values of Brownian and stretching parameters. Figures 21 and 22 show the opposite behavior. When we increase the values of $Br,$ Bejan number decreases and inclines in values of ${\delta }_{1}$ Bejan number increases. Figures 23 and 24 show the same behavior, increasing the amount of $Br$ and $d$ cause rising in $NG(\eta )$. Figures 25 and 26 scrutinize the impact of ${\delta }_{1}$ and $We$ on entropy profile. The entropy field declines for ${\delta }_{1}$ and inclines for $We$ by increasing the amount of both parameters. Streamline diagrams are shown in Figs. 27, 28, and 29 along with the different values of mixed convection $\left(\lambda \right)$ parameter. Streamline Figs. 30, 31, and 32 are incorporated along the distinct amount of magnetic parameter $\left(\beta \right)$. Numerical results of skin, Nusselt, and Sherwood numbers of various parameters against the distinct amount of magnetic parameters are discussed in tables. Table 1 demonstrates the impact of parameters on skin friction coefficient, when we increased the amount of mixed convection, Brownian, stretching parameters, and reciprocal of the magnetic Prandtl number then the values of skin friction rise. On the other hand, it is easily noticeable that the values of skin friction get down against by inclining values of buoyancy ratio and thermophoresis parameters. Skin friction changes slightly by upgrading the values of the Wessinberg number. Table 2 highlights the effectiveness of different parameters on heat transfer rate. The number of Local Nusselt tends to expand by increasing the size of Prandtl, mixed convection, Brownian, stretching parameters, and the reciprocal of the magnetic Prandtl, heat transfer rate declines when the amount of buoyancy ratio, Brownian parameters, and Lewis numbers boost. Table 3 described the mass transfer rate versus some parameters. There is a notice that local Sherwood number becomes larger quantity if buoyancy ratio, Brownian and Lewis number grow and diminishes when the mixed convection, thermophoresis, stretching, Prandtl numbers and the reciprocal of the magnetic Prandtl number getting a rise. Table 4 depicts the comparison of the present results with previously published work under some special limited cases. We found an excellent agreement of the present results with existing results. This proves the validity of the present results along with the accuracy of the numerical technique we used in this study.

**Table 1:** Variation of ${C}_{f}{\left(R{e}_{x}\right)}^\frac{1}{2}$ for distinct amounts of parameters.

$\lambda$	${N}_{r}$	$Nb$	$Nt$	$\varepsilon$	$d$	$Le$	${W}_{e}$	${\alpha }_{1}$	$n$	$Pr$	${C}_{f}{\left(R{e}_{x}\right)}^\frac{1}{2}$		
											$\beta =0.1$	$\beta =0.3$	$\beta =0.5$
0.1	0	0.1	0.1	1	1	0.1	0.1	0.1	1	9	0.020455	0.021043	0.021769
0.3											0.061256	0.063009	0.065174
0.5											0.101916	0.104819	0.108404
0.1	0.1	0.1	0.1	1	1.1	0.1	0.1	0.1	1.1	9	− 0.033985	− 0.041589	− 0.052390
	0.3										− 0.145730	− 0.170972	− 0.206937
	0.5										− 0.262142	− 0.306995	− 0.371543
0.1	0.1	0.2	0.1	1	1.2	0.1	0.1	0.1	1.2	9	− 0.013689	− 0.018545	− 0.025596
		0.3									− 0.007198	− 0.011290	− 0.017079
		0.5									− 0.002561	− 0.005943	− 0.010872
0.1	0.1	0.1	0.2	1	1.3	0.1	0.1	0.1	1.3	9	− 0.073918	− 0.086949	− 0.105362
			0.3								− 0.112849	− 0.131333	− 0.258072
			0.5								− 0.187142	− 0.216549	− 0.825600
0.1	0.1	0.1	0.1	0.5	1.4	0.1	0.1	0.1	1.4	9	− 0.593478	− 0.077532	− 0.052356
				1							− 0.033945	− 0.041543	0.778736
				1.5							0.865269	0.823401	− 0.052356
0.1	0.1	0.1	0.1	1	1.5	0.1	0.2	0.1	1.5	9	− 0.033945	− 0.041543	− 0.052356
							0.3				− 0.033945	− 0.041543	− 0.052356
							1				− 0.033945	− 0.041543	− 0.052356
0.1	0.1	0.1	0.1	1	1.6	0.1	1	0.2	1.6	9	− 0.033968	− 0.041523	− 0.051989
								0.3			− 0.033945	− 0.041353	− 0.045957
								0.5			− 0.033843	− 0.040827	− 0.049817

**Table 2:** Variation of ${Nu}_{x}{\left(R{e}_{x}\right)}^{-\frac{1}{2}}$ for distinct amounts of parameters.

$\lambda$	${N}_{r}$	$Nb$	$Nt$	$\varepsilon$	$d$	$Le$	${W}_{e}$	${\alpha }_{1}$	$n$	$Pr$	${Nu}_{x}{\left(R{e}_{x}\right)}^{-\frac{1}{2}}$		
											$\beta =0.1$	$\beta =0.3$	$\beta =0.5$
0.1	0.1	0.1	0.1	1	1	0.1	1	0.1	1	9	2.291334	2.289917	2.287892
0.3											2.295342	2.294137	2.292382
0.5											2.299314	2.298315	2.296825
0.1	0.2	0.1	0.1	1	1.1	0.1	1	0.1	1.1	9	2.280583	2.277519	2.273130
	0.3										2.269419	2.264553	2.257533
	0.5										2.245646	2.236571	2.223209
0.1	0.1	0.2	0.1	1	1.2	0.1	1	0.1	1.2	9	2.346875	2.346025	2.344801
		0.3									2.403657	2.403012	2.402076
		0.5									2.528064	2.527615	2.526962
0.1	0.1	0.1	0.2	1	1.3	0.1	1	0.1	1.3	9	2.141455	2.138960	2.135406
			0.3								1.995340	1.991693	1.986493
			0.5								1.715268	1.709107	1.709107
0.1	0.1	0.1	0.1	0.5	1.4	0.1	1	0.1	1.4	9	2.183882	2.280801	2.315940
				1							2.291334	2.289916	2.287892
				1.5							2.427953	2.422675	2.416955
0.1	0.1	0.1	0.1	1	1.5	0.1	1	0.1	1.5	9	2.291334	2.289917	2.287892
						0.3					2.102891	2.101835	2.100320
						1					1.407711	1.407646	1.407536
0.1	0.1	0.1	0.1	1	1.6	0.1	1	0.1	1.6	9	2.291334	2.289917	2.287892
								0.3			2.291354	2.290023	2.288219
								0.5			2.291380	2.290023	2.288219
**0.1**	0.1	0.1	0.1	1	1.7	0.1	1	0.1	1.7	10	2.403604	2.402174	2.400134
										14	2.787846	2.786393	2.784325
										18	3.096182	3.094728	3.092664

**Table 3:** Variation of ${Sh}_{x}{\left(R{e}_{x}\right)}^{-\frac{1}{2}}$ for distinct amounts of parameters.

$\lambda$	${N}_{r}$	$Nb$	$Nt$	$\varepsilon$	$d$	$Le$	${W}_{e}$	${\alpha }_{1}$	$n$	$Pr$	${Sh}_{x}{\left(R{e}_{x}\right)}^{-\frac{1}{2}}$		
											$\beta =0.1$	$\beta =0.3$	$\beta =0.5$
0.1	0.1	0.1	0.1	1	1	0.1	1	0.1	1	9	− 2.074730	− 2.073467	− 2.071661
0.3											− 2.078306	− 2.077232	− 2.075668
0.5											− 2.081851	− 2.080961	− 2.079632
0.1	0.2	0.1	0.1	1	1.1	0.1	1	0.1	1.1	9	− 2.065146	− 2.062415	− 2.058502
	0.4										− 2.044830	− 2.038719	− 2.029823
	0.6										− 2.022647	− 2.012324	− 1.996911
0.1	0.1	0.2	0.1	1	1.2	0.1	1	0.1	1.2	9	− 1.085121	− 1.084796	− 1.084327
		0.3									− 0.758173	− 0.758040	− 0.757847
		0.5									− 0.503354	− 0.503333	− 0.503304
0.1	0.1	0.1	0.2	1	1.3	0.1	1	0.1	1.3	9	− 3.810134	− 3.805417	− 3.798702
			0.4								− 6.426949	− 6.408015	− 6.380939
			0.6								− 7.990622	− 7.946420	− 7.882628
0.1	0.1	0.1	0.1	1	1.4	0.1	1	0.1	1.4	9	− 2.074729	− 2.073466	− 2.071662
				1.5							− 2.196546	− 2.191839	− 2.186729
				2							− 2.319789	− 2.315147	− 2.310299
0.1	0.1	0.1	0.1	1	1.5	0.1	1	0.1	1.5	9	− 2.074729	− 2.073466	− 2.071662
						0.3					− 1.432240	− 1.431597	− 1.430675
						1					− 2.074758	1.1469326	1.1466327
0.1	0.1	0.1	0.1	1	1.6	0.1	1	0.2	1.6	9	− 2.074738	− 2.073512	− 2.071807
								0.4			− 2.074758	− 2.073614	− 2.072098
								1			− 2.074833	− 2.073928	− 2.072866
**0.1**	0.1	0.1	0.1	1	1.7	0.1	1	0.1	1.7	10	− 2.173451	− 2.172179	− 2.170362
										14	− 2.508614	− 2.507326	− 2.505494
										18	− 2.773463	− 2.772182	− 2.770364

**Table 4:** Comparisons of stretching ratio $(\varepsilon )$ when ${W}_{e}=\beta ={N}_{r}=\lambda =0$ with Pop et al.^48^, Sharma and Singh^49^, and Khan et al.^50^.

$\varepsilon$	Pop et al.^48^	Sharma and Singh^49^	Khan et al.^50^	Present result
0.1	− 0.9694	− 0.9694	− 0.96939	− 0.96939
0.2	− 0.9181	− 0.9181	− 0.91811	− 0.91811
0.5	− 0.6673	− 0.6673	− 0.66726	− 0.66726
0.7			− 0.43346	− 0.43346

## Concluding remarks

This manuscript's concise report on entropy generation, induced magnetic field, and mixed convection considering pseudoplastic non-Newtonian stagnation point nanofluid flow clarifies that it is still worthy to allocate more attention to the combined convection using nanofluid on flow mass and heat transport. In our point of view, the problem is new and original. Therefore, the present research is the first attempt to use the induced magnetic field with combined convection pseudoplastic non-Newtonian (Carreau-Yasuda) nanofluid to investigate flow on mass and heat transport behavior over the elastic sheet with stagnation point. The achieved system is worked out numerically by applying a bvp4c MATLAB algorithm. The influences of effective parameters on mass and heat transport characteristics are examined. The analysis made in this article shows that the mass and heat transport rate found improved in the flow of pseudoplastic non-Newtonian nanofluid. The pseudoplastic nanofluids are applicable in all electronic devices for increasing the heating or cooling rate in them. Further, pseudoplastic nanofluids are also applicable in reducing skin friction coefficient. The main outcomes in the recent analysis are as follow.

- The Induced magnetic graph, moves downward near the boundary and rises far away with an increasing amount of the reciprocal of the magnetic Prandtl number. The induced magnetic curve expands while enhancing the amount of magnetic, mixed convection, and thermophoresis parameters where it is moved down for an increase in Brownian and stretching parameters.
- Velocity profile grows and temperature field decays for the rising amount of magnetic parameter $(\beta )$.
- Velocity declines with the increasing amount of ${\alpha }_{1}$ and Brownian parameter while temperature profile upgrades with the decrease in the values of reciprocal of the magnetic Prandtl number.
- Temperature variation diminishes with rising values of mixed convection, thermophoresis, Brownian, buoyancy ratio, and stretching parameters, on the other hand, it is noticeable that velocity profile grows with increasing values of mixed convection, thermophoresis parameter, and stretching parameter.
- Field of Bejan number decreases by inclines in $Br$ and increases by enhancing in ${\delta }_{1}$.
- Profile of entropy increase when the amount of $Br$, $We,$ and $d$ incline. Enlargement in the values of ${\delta }_{1}$ entropy field decline.

## Abbreviations

${\alpha }_{1}$: Reciprocal of the magnetic Prandtl number
${k}^{*}$: Mean absorption coefficient
$$b$$: Body forces
$$C$$: Nanoparticle concentration
$F$: Dimensionless velocity function
$${h}_{f}$$: Convection coefficient
${N}_{b}$: Brownian motion parameter
$${N}_{t}$$: Thermophoresis parameter
${Nu}_{x}$: Local Nusselt number
${C}_{\infty }$: Ambient fluid concentration
${T}_{w}$: Hot fluid temperature
$$(u, v)$$: Velocity components
$${W}_{e}$$: Weissenberg number
$\tau$: Extra stress tensor
$$d$$: Fluid parameter
${Sh}_{x}$: Local Sherwood number
$$K$$: Thermal conductivity
$\lambda$: Mixed convection parameter
${\rho }_{f}$: The density of the base fluid
${q}_{w}$: Surface heat flux
${\mu }_{\infty }$: Infinite shear rate viscosity
${A}_{1}$: First Rivilin-Ericksen tensor
$$np$$: Nanoparticle
$$\alpha $$: Thermal diffusivity
$\theta$: Dimensionless heat transfer function
$$\beta $$: Magnetic parameter
${C}_{f}$: Skin friction coefficient
${D}_{B}$: Brownian diffusion coefficient
${D}_{T}$: Thermophoresis diffusion coefficient
$$g$$: Gravity acceleration
${Re}_{x}$: Local Reynolds number
${\sigma }^{*}$: Stefan-Boltzmann coefficient
${T}_{\infty }$: Ambient fluid temperature
${U}_{w}$: Stretching sheet velocity
${U}_{\infty }$: External flow velocity
$(x,y)$: Cartesian coordinate components
$\Gamma$: Time fluid parameter
$$n$$: Power index
$$Pr$$: Prandtl number
${N}_{r}$: Buoyancy ratio parameter
${\tau }_{w}$: Surface shear stress
${q}_{m}$: Surface mass flux
${\mu }_{0}$: Zero shear rate viscosity
$$f$$: Base fluid
$$\varepsilon $$: Stretching parameter
${g}_{1}$: Dimensionless magnetic function
$\varphi$: Dimensionless concentration function
$$Le$$: Lewis number
$${\alpha }_{1}^{*}$$: Magnetic diffusivity
$$p$$: Pressure
